# Controlled Electrochemical Barrier Calculations without Potential Control

**DOI:** 10.1021/acs.jctc.3c00836

**Published:** 2023-11-07

**Authors:** Simeon D. Beinlich, Georg Kastlunger, Karsten Reuter, Nicolas G. Hörmann

**Affiliations:** † Fritz-Haber-Institut der Max-Planck-Gesellschaft, Faradayweg 4-6, 14195 Berlin, Germany; ‡ Technical University of Munich, Lichtenbergstraße 4, 85747 Garching, Germany; § Technical University of Denmark, Fysikvej 311, 2800 Kongens Lyngby, Denmark

## Abstract

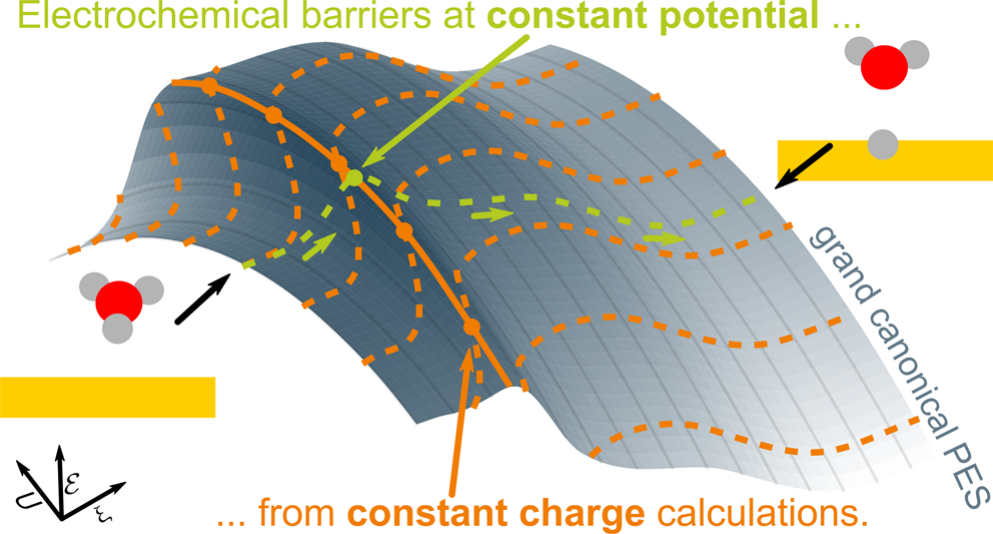

The knowledge of electrochemical activation energies under applied potential conditions is a prerequisite for understanding catalytic activity at electrochemical interfaces. Here, we present a new set of methods that can compute electrochemical barriers with accuracy comparable to that of constant potential grand canonical approaches, without the explicit need for a potentiostat. Instead, we Legendre transform a set of constant charge, canonical reaction paths. Additional straightforward approximations offer the possibility to compute electrochemical barriers at a fraction of computational cost and complexity, and the analytical inclusion of geometric response highlights the importance of incorporating electronic as well as the geometric degrees of freedom when evaluating electrochemical barriers.

## 1 Introduction

Understanding electrocatalytic activity on the atomic scale is essential for improving electrochemical energy transformation devices. While thermodynamic considerations are hereby sufficient to understand the differences between materials en gros,^1−3^ a quantitative understanding of catalytic activity, selectivity, and stability can only be achieved by knowledge of the kinetic processes, in particular electrochemical activation energies. Assessing them from ab initio calculations, e.g., via density functional theory (DFT), is, however, complicated by the compositional and configurational complexity of the interface. In addition, the reorientation of polar molecules, charge transfer, and charge rearrangement during reactions in finite computational cells at fixed electron number causes dramatic changes in the interfacial potential drop during the reaction, making it difficult to assess reaction energetics at constant potential conditions.^4−9^

Gratifyingly, most of these issues are remedied when describing the interface natively at applied electrode potential, where the number of electrons is adjusted to fulfill the constant potential boundary condition. However, such setups necessitate an appropriate electrolyte model that can counterbalance the electronic excess charges. While this poses a major obstacle for implementing such schemes in an all-explicit framework,^10−13^ corresponding calculations at finite excess charge are straightforward in implicit–explicit setups.^6,14−17^ Here, a DFT cell is coupled to a continuum solvent model that naturally provides electrolyte counter charges, and the major challenge rather lies in the availability of an efficient and stable potentiostat implementation. Most common potentiostat methods adjust the number of excess electrons *n*_e_ after each electronic self-consistent field (SCF) step in an outer loop until the electrode potential matches the target potential.^6,14,15,18−20^ More effective methods use an inner loop to adjust the potential within the SCF.^16,21^ However, both approaches often require lower SCF convergence thresholds in order to reduce numerical instabilities, leading to increased computational cost and even convergence failures, which at least can be improved with more advanced potentiostat algorithms.^20^

In this work, we present a new set of methods that can compute adiabatic grand canonical electrochemical barriers within such implicit–explicit setups with accuracy comparable to that of constant potential approaches without requiring an explicit potentiostat. The methods extend on our previous works^22−25^ that clarified that constant potential energetics can be simply obtained by the Legendre transform of an interpolated, constant charge energy landscape. In particular, we demonstrate that transition states for the proton adsorption on Au(111)—that proceeds adiabatically—^26^ as obtained from a set of constant potential Nudged Elastic Band (NEB) calculations can be fully reproduced from a set of constant charge NEB calculations.

Furthermore, we test the accuracy of a variety of computationally more efficient approximate methods based on a second-order Taylor expansion of charge and potential dependencies. The simplest method involves a single constant charge NEB calculation that fully accounts for the electronic response to the applied potential to linear order, providing the activation barrier and its linear potential dependence. More refined methods that account for second-order electronic effects and geometric responses can be obtained in a straightforward way with a handful of additional charged single point and/or additional charged NEB calculations.

In addition to offering new pathways toward computationally efficient electrochemical barrier evaluations, our detailed analysis provides a concise view on how electronic and geometric response properties affect the observed potential dependencies.

## 2 Methods

We evaluate the electrochemical barrier of a prototypical reaction—the acidic proton adsorption on Au(111) via the Volmer step
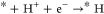
1performing DFT calculations with GPAW,^27,28^ the BEEF-vdW exchange–correlation functional,^29^ the Solvated Jellium Model (SJM)^6^ as continuum representation of the electrolyte, and the Atomic Simulation Environment (ASE).^30^ We model the transfer from a single hydronium ion surrounded by implicit solvent to a 3 × 3 Au(111) surface slab of four atomic layers thickness, of which the bottom two layers are frozen in the bulk geometry. All geometries were optimized until the force on each atom was below 0.03 eV/Å for local minima and for transition states below 0.05 eV/Å, the structures are shown in Figure S1. When used for Hessian calculations, these thresholds were reduced to 0.01 and 0.03 eV/Å, respectively. For optimization of local minima, we use the BFGS algorithm; for determining the transition states, we use the dynamic, climbing image Nudged Elastic Band (dyNEB) algorithm,^31−34^ and for Hessian calculations the vibrations module, as implemented in ASE.^30^ We perform both standard canonical DFT calculations with a fixed excess charge *q* (commonly called *constant charge* calculations) as well as grand canonical DFT calculations at fixed absolute electrode potential *U* using the built-in potentiostat of SJM (*constant potential* calculations) and show how the grand canonical energetics can equally be derived from standard canonical calculations. All computational parameters are given in the Supporting Information.

## 3 Results and Discussion

In general, the reaction path is characterized as the minimum energy path between two local minima on a potential energy surface (PES). In an electrochemical context, the relevant PES is grand canonical in the electronic degrees of freedom, where the electron number adjusts according to the externally applied potential. However, a grand canonical PES (gcPES) does not intrinsically contain more information than a constant charge, canonical PES (cPES), as both thermodynamic ensembles are directly linked to each other via a Legendre transform. Hence, as long as there is sufficient overlap, e.g., between sampled potentials or electron numbers, one can map both onto each other identically.

These ideas have already been used to study the properties of adsorbates at electrified metallic surfaces,^22−25^ and in this work, we apply them to the problem of studying electrochemical transition states. We will demonstrate how equivalent energetic information can be retrieved from transition state searches performed applying a potentiostat in order to satisfy the constant potential condition and at constant charge conditions, where the potential varies along the reaction coordinate.

**Figure 1:**
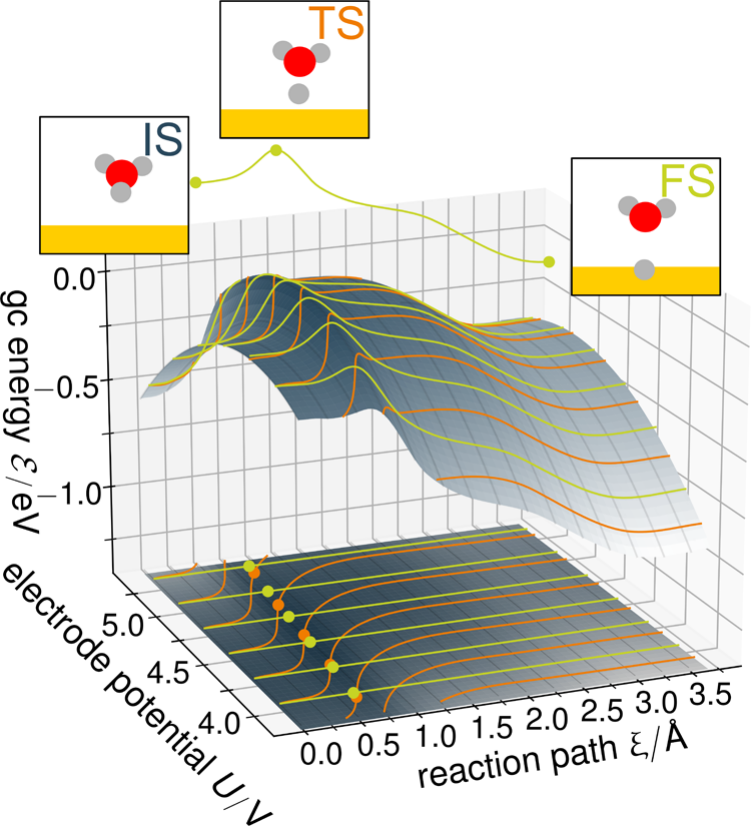
Illustration of the absolute grand canonical PES along the reaction path in dependence of absolute electrode potential *U*. Constant potential paths are shown as bright green lines, and constant charge paths are shown as orange lines. Note the slight shift of the transition state position *r⃗*_∥_^TS^ indicated by points along each path. Details of this illustration can be found in the Supporting Information.

The main difference between these two methods is the direction in which the gcPES is explored, as illustrated in Figure 1. While barrier calculations applying a potentiostat assess the gcPES along the straight constant potential lines (bright green), we can similarly explore the equivalent gcPES along the curved constant charge lines (orange) using grand canonical energies derived from constant charge calculations. As the underlying gcPES is identical, all relevant energy differences, e.g., electrochemical barriers, can be identically obtained, which we demonstrate in the present work for the example of the Volmer reaction on Au(111).

We will denote the three characteristic stationary states of our reaction path as in Figure 1 as initial state (IS, proton at water molecule in the first layer), transition state (TS), and final state (FS, Hydrogen adsorbed on a hollow hcp site).

### 3.1 Grand Canonical Energies from Canonical Calculations

Since the three characteristic states, IS, TS, and FS, are of identical composition, they represent different regions on the same PES. A direct comparison of their potential energy *E*(*q*,*r⃗*) from canonical DFT lacks the constant potential condition custom to electrochemistry. In order to introduce this latter condition, a Legendre transform needs to be performed, which transforms *E*(*q*,*r⃗*) at a given excess charge *q* to the grand canonical energy  at the respective electrode potential  by referencing any change in *q* to an external electron bath with a well-defined electrochemical potential μ̃_e_ = −e*U*:^6,11,35−38^
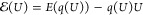
2at a given *r⃗*.

However, just as a cPES is meaningful only at a fixed number of excess electrons *n*_e_ = −*q*/e, a gcPES is meaningful only at fixed electrode potential *U*, requiring the use of a potentiostat that adjusts *n*_e_ such that *U* = const.

Considering that canonical forces *F⃗* at a constant charge *q* are identical to the grand canonical forces  at the respective constant electrode potential *U*(*q*),^39−41^
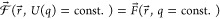
3Using the grand canonical energy and forces in combination with a potentiostat provides all relevant quantities that are necessary for common geometry optimizations or transition state search algorithms, which evaluate the PES of the system.

While this use of a potentiostat for directly returning the gcPES is appealing, to this date, only a few DFT codes provide a computationally efficient potentiostat.^6,15,17^

However, the equivalence of forces of the canonical and grand canonical PES (eq 3) implies that a geometrically stationary point r⃗*, i.e., a local extremum or a saddle point, determined in one PES is also a stationary point in the other PES.^40−42^ This allows evaluating stationary points in the cPES at certain excess charges *q*_*i*_ using structure relaxations or transition state search algorithms yielding *r⃗*_i_^*^ and *U*_i_^*^, Legendre transform their energies *E*_i_^*^ = *E*(*q*_*i*_, *r⃗*_i_^*^) to the grand canonical energies , and obtain the identical states with identical grand canonical energies as if we searched for *r⃗*_i_^*^ and  at *U*_i_^*^ in the gcPES. However, the electrode potentials *U*_i_^*^ corresponding to the excess charges *q*_*i*_ will differ for the different stationary states IS, TS, and FS.

**Figure 2:**
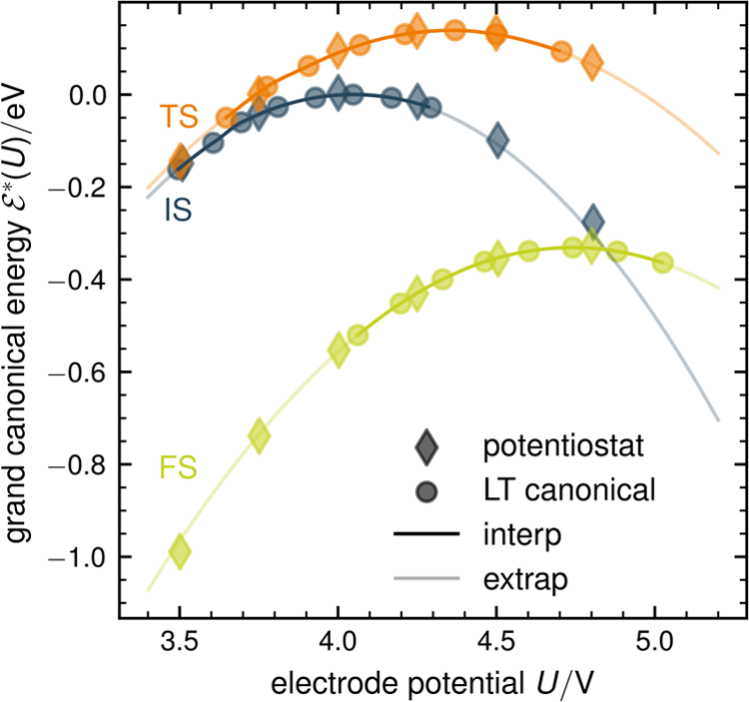
Inter-/extrapolated grand canonical energies of initial, transition, and final state as a function of absolute electrode potential derived from eight constant charge climbing image NEBs for *q* = −0.555, −0,444, ..., 0.222 e (from left to right) in comparison with potentiostat results (diamonds). Cubic Hermite spline interpolation as solid lines, quadratic extrapolation as light solidlines.

The situation is illustrated in Figure 2, where we show the grand canonical energy of IS, TS, and FS as a function of potential determined by structure relaxations and NEB calculations on the cPES at different excess charges (indicated by points) in comparison with calculations using a potentiostat (indicated by diamonds).

Both approaches yield essentially identical results, the only difference being that the potentiostat aligns all corresponding data points vertically at the chosen potentials, thus allowing us to directly compute energy differences, e.g., reaction energies or kinetic barriers, in a point-wise manner.

### 3.2 Approaches Based on Multiple Canonical NEB Calculations

The detailed procedures of the presented methods are outlined in the Supporting Information.

#### 3.2.1 Inter-/Extrapolation Including Geometric Response

Evidently, instead of using a potentiostat, we can equally interpolate the grand canonical energy of a geometrically stationary state  by choosing an appropriate interpolation method in the potential range that is fully covered by the explicitly calculated data points at varying charge *q*_*i*_. It might additionally benefit from the first derivative of the grand canonical energy of a stationary state, which is given by the excess charge^41^
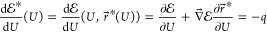
4since  and  for stationary points:
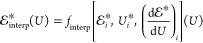
5In Figure 2, we show the interpolation using a cubic Hermite spline, taking into account the slope as solid lines. It yields excellent agreement with the data from the potentiostat calculations—especially considering that canonical and potentiostat-based results are derived from separate geometric relaxations and NEBs. Given a fine enough grid of excess charges *q*_*i*_, the specific choice of an interpolation function is hereby of no great importance.

This interpolation-based method can, in principle, re-create the same results as a calculation using a potentiostat and requires multiple NEB calculations in order to cover the desired potential range and resolution. Considering that the analysis of transition paths across a range of electrode potentials, which is typically of central interest, necessitates similar interpolation across a set of constant potential results, the central difference between the present canonical-based and established potentiostat-based barrier calculations lies only in the sequential ordering of interpolation and Legendre transform.

One drawback of the interpolation-based method is its limitation to the potential range that is explicitly covered by the chosen *q*_*i*_. However, as evident from Figure 2, the potential dependence of the grand canonical energy of a stationary state is not very complex and typically well described by a parabolic capacitor-like expression recognized and exploited in a wide range of previous works considering the grand or the canonical ensemble.^5,7−9,22−25,38,43−47^ In line with the common practice in the field,^48^ we refer to the second-order expansion coefficient as capacitance, noting that it does not directly relate to the experimentally observable capacitance. Considering that  always exhibits a maximum at the potential *U*_PZC_^*^ where the given state exhibits zero excess charge (*q*_PZC_ = 0 in eq 4, cf. ref (49)), we can write
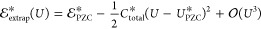
6where , *U*_PZC_^*^, and the capacitances *C*_total_^*^ can for now be considered simple, constant fit parameters unique to every stationary state describing the parabolic potential dependence of  along r⃗*(*U*). Using a parabolic model function allows a physically sensible and accurate extrapolation to potentials outside the range explicitly calculated, while the accuracy difference to higher-order interpolation methods, e.g., a cubic Hermite spline interpolation, is negligible, as shown in Figure 2. Keep in mind, however, that other systems might exhibit a more complex potential dependence, where the capacitance is a potential-dependent quantity.

As a word of caution, besides the deviations caused by higher-order contributions at potentials far from *U*_PZC_^*^, a special case might arise when a stationary state becomes energetically unstable, dropping into a second nearby local minimum/saddle point (which translates into a discontinuity of r⃗*(*U*)), leading to an abrupt change in the grand canonical energetics. In such a case of a competing state, we would have to consider that alternative state in the same manner but separately in the energetics. We emphasize, however, that these special cases are not specific to our method but equally have to be considered in the potentiostat method or any other electrochemical barrier method.

### 3.3 Approaches Based on a Single Canonical NEB Calculation

The parabolic extrapolation formula eq 6 exhibits only three free parameters, which can be obtained by performing multiple, but only a few, charged NEB calculations. However, if we knew *C*_total_^*^, then it would be possible to parametrize eq 6 directly from only a single, converged, constant charge NEB calculation. As we demonstrate in the following, *C*_total_^*^ can indeed be approximated at various levels of detail and accuracy without performing additional NEB calculations. At the crudest level, we can approximate *C*_total_^*^ by a single value *C*_single_ that is constant and independent of the considered state of the surface. In a first more refined approach, we approximate *C*_total_^*^ for each of the stationary states IS, FS, and TS by only considering the electronic response with , i.e., at r⃗* = const. The most accurate method further includes the effect of geometric response, which can be expressed as a geometric capacitance contribution where *C*_total_^*^ = *C*_el_^*^ + *C*_geom_^*^.^41^

Note also that while it is reasonable to perform the geometric optimizations—relaxations and TS search—at*q*_PZC_ = 0, one can determine it equally at any given *q*, e.g., if some states are not accessible (stable) at zero excess charge (see the Supporting Information).

#### 3.3.1 Single Capacitance Approximation: SC

**Figure 3:**
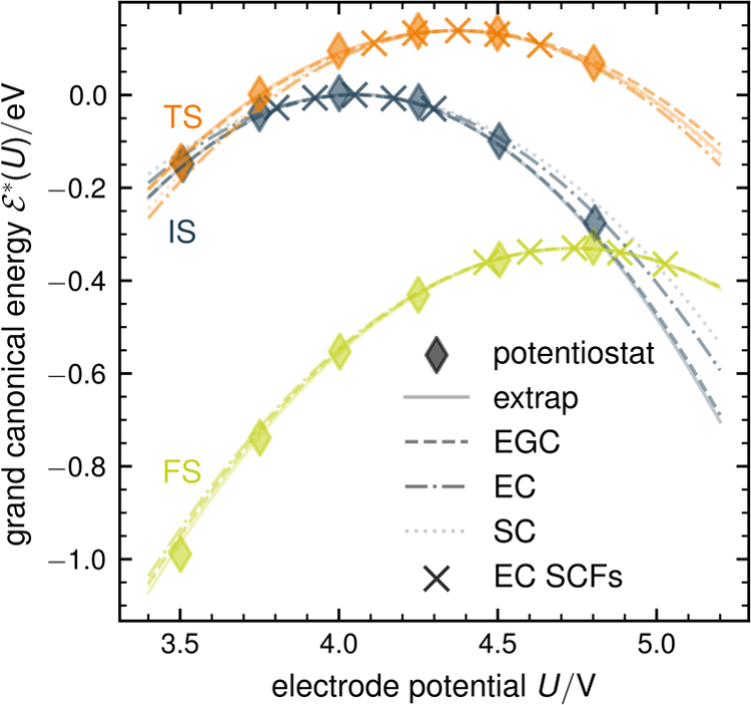
Grand canonical energies of IS, TS, and FS in the SC, EC, and EGC approximation (dotted, dashed-dotted, and dashed lines) in comparison to extrapolated (light solid lines) and reference potentiostat results (diamonds). The electronic SCF calculations used for determining the electronic capacitance of the EC approach are shown as crosses. While SC and EC results lead to reasonable agreement with the reference data, the EGC approximation agrees nearly perfectly with the extrapolated and reference results. Note the large contributions of geometric effects for IS and TS (difference between EC and EGC (dashed-dotted and dashed lines)).

Let us consider now the crudest of approximations, where we assume *C*_total_^*^ ≈ *C*_single_ to be identical for all considered states and potentials. While using a single, invariable capacitance seems oversimplistic, a corresponding approximation is very common in electrochemical contexts, e.g., in barrier calculations based on the charge extrapolation^5^ or the mean potential method^7−9^ or in the description of adsorption under applied potential conditions within an effective dipole-field approximation.^43^ Considering that our explicitly determined values for *C*_total_^*^ are 24.0 μF/cm^2^ (IS), 16.7 μF/cm^2^ (TS), and 18.6 μF/cm^2^ (FS), the SC approximation seems not overdramatic for the present system. This is evidenced by comparing the grand canonical reference results with the predictions obtained from the single canonical NEB, single capacitance (SC) approximation (Figure 3, diamonds and dotted lines, respectively). The agreement is certainly impressive, considering that the reference results are obtained from multiple, grand canonical, constant potential NEB calculations while the SC results are obtained from a single canonical NEB at *q*_PZC_ = 0 and with *C*_single_ = 18.2 μF/cm^2^ determined from a finite difference evaluation of  based on a set of five charged SCF calculations (*q*_*i*_ = ± 0.222, ± 0.111, 0.0 e) of a Au(111) surface without a proton but only with a water molecule that is geometrically fixed at r⃗*(*q*_PZC_ = 0) = const. As a final remark, if *C*_single_ is not explicitly computed but assumed (e.g., based on experimental data), the SC method can equally be applied to infer constant potential barriers, only based on a single canonical, *q*_PZC_ = 0 NEB calculation, which removes even the necessity of an appropriate counter charge model (e.g., implicit solvent environment).

#### 3.3.2 Electronic Capacitance Approximation: EC

The approximation following SC in complexity is straightforward: instead of assuming identical capacitances for all three states, we approximate them independently by probing the purely electronic response of each state. This approach considers only the explicit dependence of  on *U* while keeping r⃗* = *r⃗*_PZC_^*^ fixed at the geometry of the stationary point at *q*_PZC_ = 0. Mathematically, this corresponds to a second-order expansion only in the electronic degrees of freedom around each state’s PZC:
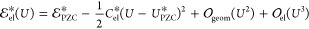
7where *C*_el_^*^ is considered constant. Practically, this equation is parametrized by performing first a single geometry optimization at constant *q*_PZC_ = 0, followed by charged electronic SCF calculations (*q*_*i*_ = ± 0.222, ± 0.111 e) at fixed geometry for each of the states of interest, which yields all necessary information on the electronic response properties. The resulting EC results are shown as crosses and dashed-dotted lines in Figure 3. The missing geometric response leads to larger deviations at potentials further away from each *U*_PZC_^*^, which leads us to the final set of approximate, single-shot barrier methods that include further the geometric response up to second order.

#### 3.3.3 Electronic and Geometric Capacitance Approximation: EGC

Until now, we either approximated the potential-dependent grand canonical energy of a stationary state  ignoring the individual geometric response r⃗*(*U*) of each stationary state (SC and EC approximation), or we directly sampled and inter-/extrapolated *U*_i_^*^ and  with accurate geometric response *r⃗*_i_^*^ by performing multiple geometry optimizations at various *q*_*i*_.

We can, however, also follow an expansion-type approach for the geometric response, i.e., evaluate how *r⃗*(*U*) responds to a change in potential *U* analytically. For this, we expand the grand canonical energy  in both *U* and *r⃗* around a stationary point, e.g., (*U*_°_^*^, *r⃗*_°_^*^), yielding a 3*N* + 1-dimensional parabolic expression  that is accurate up to second order in *U* and *r⃗* around *U*_°_^*^, *r⃗*_°_^*^. The stationarity condition  then yields the linear geometric shift of *r⃗*(*U*) and finally . Due to their length and general importance, the mathematical details of this analysis are reported separately in ref (41); a shorter summary is given in the Supporting Information.

Such derived potential dependence of the grand canonical energy of a stationary point is given by
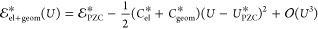
8
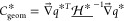
9where we consider an expansion around the PZC, without loss of generality (see the Supporting Information). Here, *C*_el_^*^, as above, denotes the purely electronic capacitance,  the inverse of the 3*N* × 3*N*-dimensional, grand canonical Hessian and ∇⃗q* the 3*N*-dimensional gradient of the excess charge in the 3*N* spatial degrees of freedom—all properties being evaluated at *U*_PZC_^*^ and *r⃗*_PZC_^*^ and considered constant (see the Supporting Information for a discussion of the potential dependence of *C*_geom_^*^). We can obtain the required properties from a common (grand canonical) Hessian calculation, i.e., a finite-differences evaluation of the changes of forces  and excess charge  caused by a displacement of the system along one of the 3*N* atom coordinates *r*_*j*_ while using a potentiostat to maintain constant potential conditions. The value of ∇⃗*q* can equally be determined as —the change of force caused by a change in potential *U* (see the Supporting Information or refs^9,41^).

However, following the spirit of this work, we rewrite this expression into purely canonical quantities and thus parametrize it based on constant charge calculations only. For this, we make use of the general relation between the grand canonical Hessian  and the canonical Hessian *H*:^41^
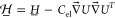
10where the gradient of the electrode potential ∇⃗*U* is closely related to its grand canonical counterpart—the gradient of the excess charge ∇⃗*q*—via:^41^
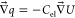
11Note that the electronic capacitance *C*_el_ is identical in the canonical and grand canonical ensemble and that all quantities depend on the respective independent variables *r⃗* and *U* or *q*.^41^ Similar to the grand canonical case, performing a common canonical Hessian calculation yields both *H* and ∇⃗*U*, i.e., all quantities needed in order to derive  and ∇⃗*q*, and finally, *C*_geom_^*^ via eqs 11, 10, and 9. For an exhaustive mathematical derivation, explanation, and discussion, we encourage the interested reader to follow the derivation in the Supporting Information or ref (41).

The corresponding energetics using *C*_geom_^*^ derived from purely canonical calculations at *q*_PZC_ = 0 are plotted in Figure 3 as dashed lines. The analytically derived, Hessian-based result  and the fitted, parabolic expression  agree perfectly, reflecting the relevance of accounting for the potential-induced geometric displacements. Instead of performing multiple NEB calculations, we can equally derive the quadratic potential dependence, including geometric effects, by performing only a single NEB calculation in combination with additional single point calculations to obtain the Hessian and *C*_el_. As a consequence, we can get results that are essentially as good as the multi-NEB approach at a dramatically reduced effort and computational cost. The impact of including geometric effects is in fact even more significant when considering the actual activation energies, i.e., the energetic difference between transition state and resting states, as we show in the following.

### 3.4 Kinetic Barriers

Activation energies between a resting state α = IS, FS and the transition state TS, i.e., for the forward and backward reaction, are given by the respective energy differences . For simplicity, here, we only consider the states discussed until now, i.e., the forward barrier with respect to the proton in the interfacial water layer. The interested reader finds the same analysis for an initial state that considers the proton in the bulk electrolyte in the Supporting Information.^18,40^

**Figure 4:**
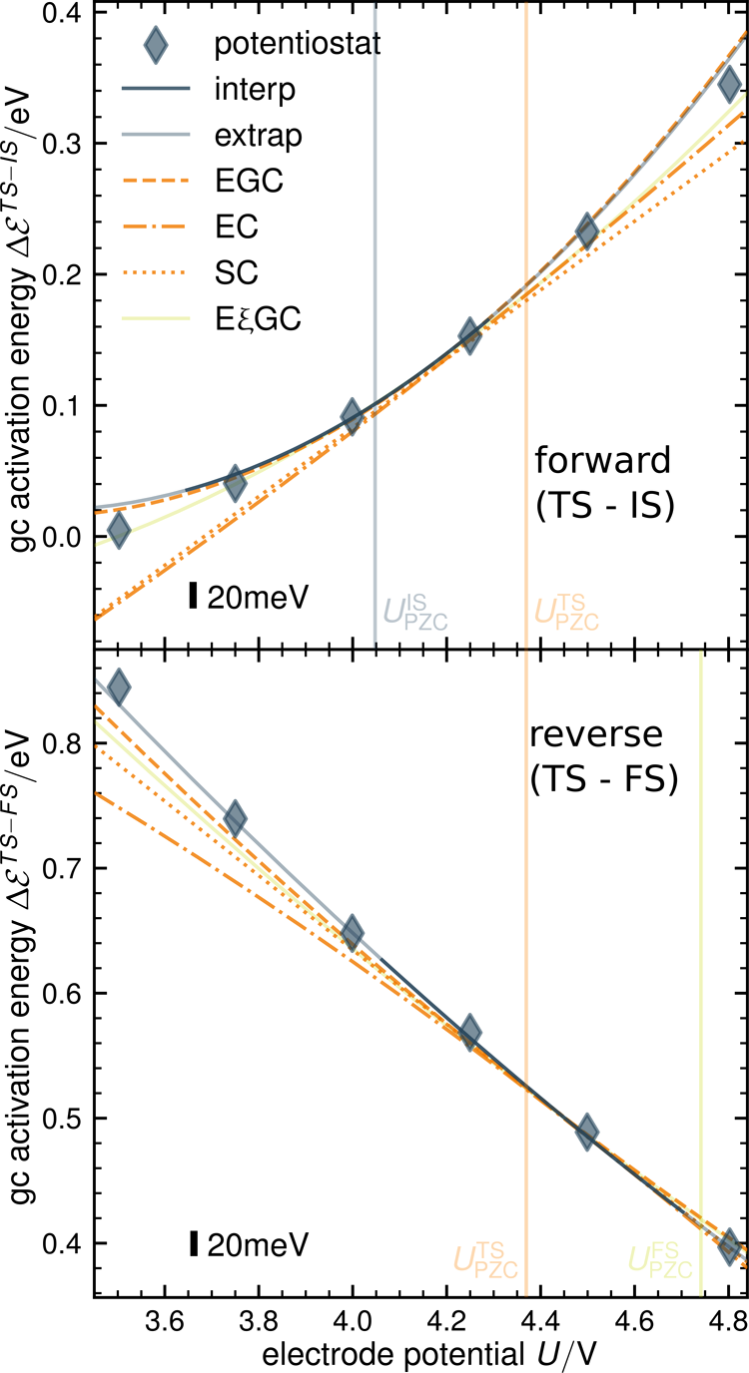
Comparison of kinetic forward and reverse barriers in dependence of the absolute electrode potential. Multiple NEB calculations-based methods (blue solid lines): interpolation (dark), extrapolation (light). Single-NEB methods (orange lines): EGC (electronic + geometric, dashed), EC (electronic only, dashed-dotted), and SC (single capacitance, dotted). Reference potentiostat data is shown as dark blue diamonds. Note the strong influence of geometric effects, i.e., the difference between EGC (dashed) and EC approximation (dashed-dotted), which are, to a large extent, originating from contributions along the reaction path ξ (EξGC, bright green lines). The PZCs of the relevant states *U*_PZC_^*^, which are accurately probed by the *q* = 0 NEB used for SC, EC, EGC, and EξGC, are indicated by light vertical lines.

First, we compare the inter- and extrapolated results obtained from multiple canonical NEB calculations using a parabolic fit, which are shown in Figure 4 as dark and light blue solid lines, with the potentiostat-derived reference results (diamonds).

The excellent agreement between these results, with maximum deviations smaller than 20 meV, demonstrates that the quadratic extrapolation of constant charge calculations can already re-create the nonlinear barrier changes with potential. The nonlinearity originates from the TS shifting toward the energetically less favored state (in the case of the forward barrier, the IS for low potentials, and vice versa for the reverse barrier).^18,40,50,51^

Of the approximate expansion-type methods based on a single canonical NEB calculation, unsurprisingly, the Hessian-based EGC method performs the best (dashed orange lines). It essentially re-creates the extrapolated results that require multiple canonical NEB calculations, indicating that the individual electronic and geometric responses of the respective states determine the nonlinear potential dependence of the barrier.

When we include only the pure electronic response individually for all involved states (EC approximation) or even only a single, constant capacitance (SC approximation), we obtain a slightly different picture. Here, predictive accuracy is only obtained in close vicinity of the potentials that are explicitly probed by the constant charge NEB (vertical lines in Figure 4). In particular, the methods fail to reproduce the nonlinear potential dependence across the studied potential window. While the SC approximation can only describe a linear potential dependency by construction, since the identical quadratic contributions cancel out, the nonlinear potential dependence obtained within the EC approximation is markedly different from the actually observed trend. This is especially evident for the backward, TS-FS barrier (cf. Figure 4). This clarifies that the seemingly more refined EC approximation that accounts for differences in the purely electronic response at IS, FS, and TS does indeed not improve upon the simplistic approach with only one single, constant electronic capacitance. As such, it highlights the importance of accounting not only for the electronic but also for the geometric response systematically, as also pointed out by others before.^48^

We can even go a step further and evaluate the origin of the nonlinear potential dependence of the activation energies by dissecting it into its various contributions. Within the present quadratic approximations, we obtain for the linear and quadratic potential dependencies
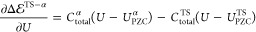
12
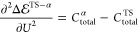
13Hence, within the SC approximation, where *C*_total_^α^ = *C*_total_^TS^ = *C*_single_, the nonlinear term vanishes and the linear term is simply given by the differences in the potentials of zero excess charge *C*_single_Δ*U*_PZC_^TS−α^, identical to the potential dependence in an effective dipole-field approximation.^43^ Already, with the simplest method, we can thus determine the linear potential dependence, in essence, the electrochemical symmetry factor relative to the state α.

As evident from eq 13, nonlinearities originate from capacitance differences between the considered states, which can be split into electronic and geometric contributions:
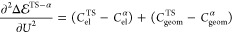
14Note that the local minima α and the transition state differ in one significant property: along the reaction path, the transition state is a local maximum. Following this idea, let us consider a case where geometric response occurs only along the transition path coordinate ξ, i.e., a purely one-dimensional problem. In this case, we find that
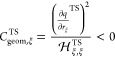
15since the reaction path ξ is a normal mode of the grand canonical Hessian with a negative eigenvalue  (cf. ref (41)). In contrast, *C*_geom,ξ_^α^ > 0 for the local minima IS and FS, since  is positive. These opposing contributions are the reason why the geometric response leads to a lower total capacitance in comparison to the purely electronic description for the TS (the dashed EGC approximation lies above the dotted EC in Figure 3) but a higher total capacitance for the IS and the FS (EGC lies below the EC in Figure 3). These considerations clarify that there is a distinct difference in the geometric response of resting states and transition states, which drive the overall curvature  toward positive values (cf. Figure 4) leading to the observed convex nonlinear potential dependence of the activation barrier.

In order to assess the validity of this simplified, one-dimensional analysis of the geometric influence on electrochemical barriers, we reevaluate the EGC approximation while only taking the geometric response along the reaction path ξ into account. Essentially, we analytically include the effect of the potential-dependent geometric shift of the transition state along the reaction path, as indicated in the inset of Figure 1 by points. The EξGC results are plotted as green lines in Figure 4, which demonstrates that, indeed, most of the geometric contributions originate from the response along the reaction path, at least for the studied system.

We want to emphasize that since ξ represents an eigenmode of the grand canonical Hessian at the TS, we can obtain *C*_geom,ξ_^TS^ either by diagonalizing  and selecting the respective component to evaluate eq 15, or, even simpler, by determining the local curvatures of  along the reaction path at the transition state. Simplifying even more by neglecting the differences between the direction of canonical and grand canonical normal modes, we can calculate *C*_geom,ξ_^TS^ from only a single canonical NEB calculation (see the Supporting Information). The latter method thus removes the necessity to compute the full Hessians and can instead be directly evaluated from the already performed canonical NEB calculation, as long as the resolution along the path is sufficiently dense. As a result, this approximation makes the most efficient use of all of the data already available from a single NEB calculation.

## 4 Conclusions

In this work, we derive accurate electrochemical constant potential activation energies from common, canonical constant charge calculations, in principle removing the need for using a potentiostat. This is achieved by exploiting the special properties of geometrically stationary points in the grand canonical and canonical PES.

Furthermore, we show that the grand canonical energetics of the relevant states of the Volmer step on Au(111) are described with excellent accuracy using a second-order polynomial in the potential *U*—implying that the entire potential-dependent energetics can be derived from a single constant charge NEB calculation—as long as the second-order expansion coefficient, the capacitance *C*_total_, is determined accurately enough.

Leveraging this, we present a set of highly efficient methods at various degrees of accuracy: from a rough estimate based on a single, state-independent capacitance, capturing already the linear potential dependence of electrochemical barriers, via inclusion of the state-specific but purely electronic response to finally an accurate analytical incorporation of all geometric degrees of freedom to second order, which essentially re-creates the nonlinear potential dependence of electrochemical barriers obtained from multiple NEB calculations.

Our analysis furthermore highlights the central importance of considering the geometric response, in particular along the reaction path coordinate in the case of electrochemical barriers. More generally, we show that the geometric response of stationary points can be mapped onto a geometric capacitance—with contributions at transition states opposite to that at local resting states. These analytic second-order results remain valid in the vicinity of the sampled data, even for other systems where higher-order contributions might become more relevant.

Besides the practical use of the presented methods, e.g., efficient high throughput studies, this work provides a detailed qualitative and quantitative understanding on the importance of geometric effects in first-principles simulations of electrochemical interfaces.

## Supporting Information Available

Atomic structures; computational details; procedures; absolute one-dimensional grand canonical PES along the reaction path, including the reference state; derivation of geometric capacitance and grand canonical Hessian; potential dependence of stationary point geometries; potential dependence of geometric capacitance; and potential dependence of normal modes, Hessian eigenvalues, and vibrational energy contributions (PDF)
