# Supplementary material for: Controlled Electrochemical Barrier Calculations without Potential Control
Source: J Chem Theory Comput. 2023 Nov 7;19(22):8323–31. doi: 10.1021/acs.jctc.3c00836 (PMC10688182; doi:10.1021/acs.jctc.3c00836)
Supplement: Supplementary file 1 — ct3c00836_si_001.pdf [file ct3c00836_si_001.pdf]

Supporting Information: Controlled Electrochemical Barrier Calculations without Potential Control

Simeon D. Beinlich,*,†,‡ Georg Kastlunger,¶ Karsten Reuter,† and Nicolas G. Hörmann†

†*Fritz-Haber-Institut der Max-Planck-Gesellschaft, Faradayweg 4-6, 14195 Berlin, Germany*

‡*Technical University of Munich, Lichtenbergstr. 4, 85747 Garching, Germany*

¶*Technical University of Denmark, Fysikvej 311, 2800 Kongens Lyngby, Denmark*

E-mail: *beinlich@fhi.mpg.de

1 Proton transfer to Au(111) - atomic structures and potential dependence

To demonstrate our methods we model the proton transfer from a single hydronium ion surrounded by implicit electrolyte to a Au(111) surface. We find that the proton is transferred first to an (energetically unstable) atop position of the neighboring metal atom and subsequently comes to rest in the nearby hollow adsorption site. The structures of initial, transition, and final state (IS, TS, FS) and their dependency on electrode potential U are shown in Fig. S1. While IS and TS respond to changes in the electrode potential, there is vanishing response for the FS. We use the position along the $3N$-dimensional path of the reaction from IS to FS as reaction coordinate as implemented in the Atomic Simulation Environment (ASE)[1] Nudged-Elastic-Band (NEB) package.

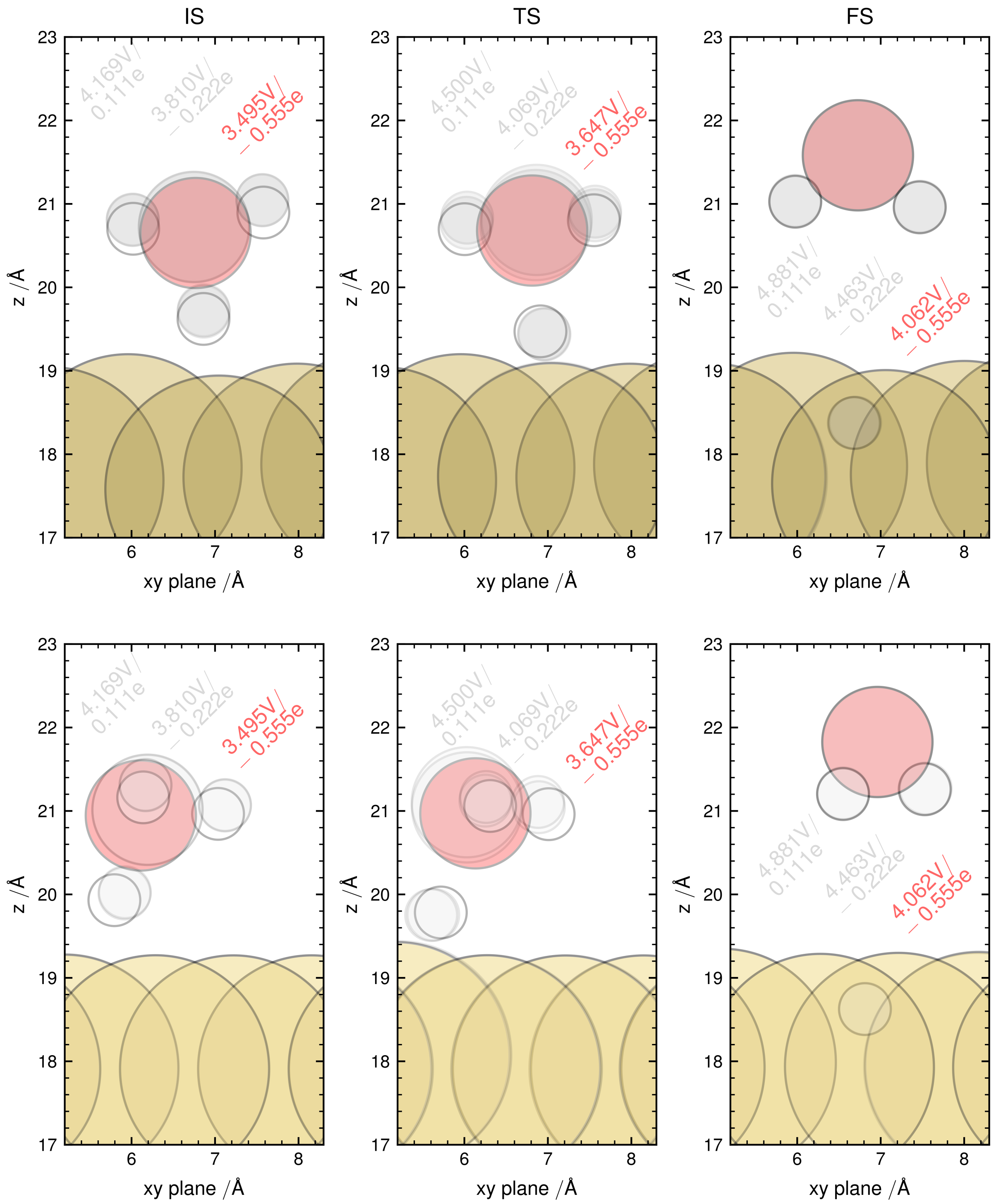

Figure S1: Atomic structures of IS, TS, and FS (left, middle, and right panels) and their dependency on electrode potential U (inserts) indicated by gray overlays from two different perspectives (top and bottom panels). Also given as inserts are the respective excess charges q.

2 Computational Details

All density functional theory (DFT) calculations were performed using the ASE[1] and the DFT code GPAW[2,3] in combination with the Solvated Jellium Model (SJM)[4] for simulating electrochemical interfaces. We employ the BEEF-vdW exchange-correlation functional[5] and a real space grid with a spacing of below 0.17 Å. This results in a $48 \times 48 \times 192$ grid for the studied (hexagonal) 3×3 Au(111) slabs with a thickness of 4 atomic layers, the bottom two of which were frozen in the bulk geometry that was optimized resulting in a lattice constant of 4.275 Å. The vacuum layer between the slabs has a thickness of at least 20 Å. The Brillouin zone is sampled using an even-numbered Γ-centered Monkhorst-Pack grid with a maximum distance of the K-points of $0.15\,\text{Å}^{-1}$ in lateral directions and a single K-point in z-direction yielding a $6 \times 6 \times 1$ K-point grid. For the bulk calculation we use a $18 \times 18 \times 18$ k-point grid. Pulay Mixing is applied (linear mixing coefficient $\beta = 0.05$, at maximum with 3 previous densities, and a weight of 50.0 for the metric) and Fermi-Dirac smearing on the occupations with a width of 0.1 eV.

The electrolyte is modelled by an implicit solvent (linear dielectric constant of 78.36, surface tension of $1.1484 \cdot 10^{-3}\,\text{eV}/\text{Å}^2$) where the cavity of the quantum-mechanical region is defined by an effective *power12potential* with the default potential strength (0.18 eV) and a temperature of 298.15 K. We scaled the Van-der-Waals radii of the gold atoms by a factor of 1.08 yielding a capacitance of the clean Au(111) slab of $19\,\mu\text{F}/\text{cm}^2$ and a PZC of 4.7 V on an absolute scale which is in close agreement to the experimental values of $\approx 15 - 30\,\mu\text{F}/\text{cm}^2$[6] and ≈ 4.9 V[7,8]. The jellium counter charge in the electrolyte above the slab used for both potential-controlled grand canonical as well as charged canonical calculations is inserted at a distance of 3 Å to the highest atom and 1 Å below the dipole-correction layer. The deviation of the electrode potential from the target potential in potential-controlled calculations is converged to below 5 mV and to 1 mV for the grand canonical Hessian calculations discussed below.

Structure optimizations are performed with the BFGS algorithm (convergence of forces below 0.03 eV/Å) and transition state searches with the dynamic Nudged-Elastic-Band (dyNEB) method,[9–11] both as implemented in ASE. The dyNEBs are first pre-converged then refined using

the climbing-image method[12] (forces below $0.05\,\mathrm{eV/Å}$). When used as the central point for Hessian calculations, we converged until the atomic forces at resting states are below $0.01\,\mathrm{eV/Å}$ and at transition states below $0.03\,\mathrm{eV/Å}$. Hessian calculations use a displacement of $0.1\,\mathrm{Å}$, and the central difference method between positive and negative displacement directions. We performed the analyses both taking into account all non-metal atoms and all metal atoms of the first layer as well taking only into account the Au atom involved in the reaction and the non-metal atoms yielding essentially identical results.

For all figures showing absolute energies, we choose the grand canonical energy of the initial state at $q = 0$ as the reference energy.

3 Procedures

In the following we give a short schematic description of the various methods discussed in the main text.

3.1 Methods based on multiple NEB calculations

Potentiostated constant potential calculations

- Perform all calculations at constant potential conditions using a potentiostat.
- For every potential U_i of interest perform the following steps:
  - Relax the initial and final state (IS and FS) to their resting positions.
  - Converge a dyNEB transition state search from IS to FS, first without the climbing image algorithm and subsequently with the climbing image algorithm.
  - Obtain the grand canonical energy $\mathscr{E}_i^*$ and the calculated excess charge q_i^* for IS, TS, and FS. If the grand canonical energy is not directly provided by the DFT code, derive it from the canonical energy E_i^*, the potential U_i and the excess charge q_i^* for every state using Eq. 2 in the main text.

– The difference in energies $\mathscr{E}_i^*$ between the respective states yields the forward and reverse activation barriers $\Delta\mathscr{E}_i^{\mathrm{TS-IS}} = \mathscr{E}_i^{\mathrm{TS}} - \mathscr{E}_i^{\mathrm{IS}}$ and $\Delta\mathscr{E}_i^{\mathrm{TS-FS}} = \mathscr{E}_i^{\mathrm{TS}} - \mathscr{E}_i^{\mathrm{FS}}$ at potential U_i.

Interpolation / Extrapolation of grand canonical energies from canonical calculations

- Perform all calculations at constant charge conditions.
- For every excess charge q_i of interest perform the following steps:
  - Relax the initial and final state (IS and FS) to their resting positions.
  - Converge a dyNEB transition state search from IS to FS, first without the climbing image algorithm and subsequently with the climbing image algorithm.
  - Obtain the canonical energy E_i^* and the calculated electrode potential U_i^* for IS, TS, and FS.
  - Derive the grand canonical energy of each state $\mathscr{E}_i^*$ at potential U_i^* via $\mathscr{E}_i^* = E_i^* - q_i U_i^*$ for every state (Eq. 2 in the main text).
- Perform an inter- or extrapolation of $\mathscr{E}_i^*$ in dependence of U_i^* for each of the states IS, TS, and FS, yielding a function $\mathscr{E}^*(U)$. If desired, use the slope $\frac{\mathrm{d}\mathscr{E}}{\mathrm{d}U}(U_i^*) = -q_i$ as additional information for inter-/ extrapolation.
- Derive the forward and reverse activation barriers as a function of U using $\Delta\mathscr{E}^{\mathrm{TS-IS}}(U) = \mathscr{E}^{\mathrm{TS}}(U) - \mathscr{E}^{\mathrm{IS}}(U)$ and $\Delta\mathscr{E}^{\mathrm{TS-FS}}(U) = \mathscr{E}^{\mathrm{TS}}(U) - \mathscr{E}^{\mathrm{FS}}(U)$.

3.2 Methods based on s single NEB calculations

All of the single NEB based methods follow the same procedure:

- Perform all calculations at constant charge conditions at excess charge q_0.
- Relax the initial and final state (IS and FS) to their resting positions.

- Converge a dyNEB transition state search from IS to FS, first without the climbing image algorithm and subsequently with the climbing image algorithm.

- Obtain the canonical energy E_0^* and the calculated electrode potential U_0^* for IS, TS, and FS.

- Derive the grand canonical energy $\mathscr{E}_0^* = E_0^* - U_0^* q_0$ for every state (Eq. 2 in the main text).

- Derive the respective approximation for the total capacitance C_{total}^* associated with each method as described below.

- If using $q_0 = 0$, i.e. $U_0^* = U_{\text{PZC}}^*$, the grand canonical energy $\mathscr{E}^*(U)$ of each state in dependence of U is given by (cf. Eqs. 6 of the main text):

$$\mathscr{E}^*(U) = \mathscr{E}_{\text{PZC}}^* - \frac{1}{2} C_{\text{total}}^* (U - U_{\text{PZC}}^*)^2 \quad .$$

  If using $q_0 \neq 0$, either compute $U_{\text{PZC}}^* = U_0^* - \frac{q_0}{C_{\text{total}}^*}$ (since $q^*(U) = C_{\text{total}}^*(U - U_{\text{PZC}}^*)$, Eq. 4 in the main text) and use aboves formula or directly use that in a parabolic approximation the grand canonical energy $\mathscr{E}^*(U)$ of each state in dependence of U is given by:[13]

$$\mathscr{E}^*(U) = \mathscr{E}_0^* - q_0(U - U_0^*) - \tfrac{1}{2} C_{\text{total}}^* (U - U_0^*)^2 \quad .$$

The different methods – single capacitance approximation (SC), electronic capacitance approximation (EC), electronic + geometric capacitance approximation (EGC), and the electronic + geometric capacitance approximation along the reaction path (EξGC) only differ in how the capacitance is derived:

Single capacitance approximation (SC)

- Use an appropriate guess for C_{total}^*, e.g. compute it for a certain state of the system. In order to do so, perform electronic single point calculations at several charges q_i for the chosen state and obtain the electrode potentials U_i^*.

- Derive the capacitance as the slope of a linear fit of q_i with respect to U_i^*:

$$q_i \propto C^*_{\mathrm{total}} U_i^* \quad .$$

Electronic capacitance approximation (EC)

- For each of the considered states IS, TS, and FS of the system perform electronic single point calculations at several charges q_i and obtain the electrode potentials U_i^*.
- For each state, derive the capacitance as the slope of a linear fit of q_i with respect to U_i^*:

$$q_i \propto C^*_{\mathrm{total}} U_i^* \quad .$$

Electronic + geometric capacitance approximation (EGC) The total capacitance is given by the sum of electronic and the geometric capacitance contributions C^*_{el} and C^*_{geom}, respectively (cf. Eq. 8 in the main text or Ref. 13):

$$C^*_{\mathrm{total}} = C^*_{\mathrm{el}} + C^*_{\mathrm{geom}} \quad . \tag{1}$$

Derive the electronic contribution C^*_{el} as described in the EC approximation above.

For the geometric contribution C^*_{geom} (using purely canonical calculations) perform the following steps for each of the considered states IS, TS, and FS:

- Compute the canonical Hessian $\underline{H}^*$ and the corresponding gradient in electrode potential $\vec{\nabla} U^*$ of the relevant part of the system (e.g. the metal atoms in the first layer of the electrode and any non-metal species) at the excess charge q_0. For this, choose the optimized structure of each state as the center structure (from relaxation or from the transition state search). Then, for all of the relevant coordinates r_i, i.e. for each of the coordinates of the relevant atoms:
  - Displace the system along r_i by a certain amount d in both positive and negative directions $(+), (-)$.

– Perform an electronic single point calculation for both displacements.

– Obtain the resulting forces $F_j^{+,-}$ along each of the relevant coordinates r_j of the system and obtain the resulting electrode potentials $U^{+,-}$ caused by the positive and the negative displacement.

– The value $H^*_{i,j}$ of the Hessian and the value of $(\overrightarrow{\nabla} U)^*_i$ are given by:

$$H^*_{i,j} = -\frac{F_j^+ - F_j^-}{2d}$$
$$(\overrightarrow{\nabla} U)^*_i = \frac{\partial U}{\partial r_i}^* = \frac{U^+ - U^-}{2d} \quad .$$

- Hessians, by definition, are symmetric matrices. In order to eliminate numerical noise, symmetrize the Hessian by replacing $H^*_{i,j}$ with $\frac{1}{2}(H^*_{i,j} + H^*_{j,i})$ from the just derived values.

- Compute the grand canonical Hessian $\underline{\mathscr{H}}^*$ via Eq. 10 in the main text:

$$\mathscr{H}^*_{i,j} = H^*_{i,j} - C^*_{\mathrm{el}} \frac{\partial U}{\partial r_i}^* \frac{\partial U}{\partial r_j}^* \quad .$$

  using the electronic capacitance derived above for the considered state.

- Calculate the inverse of the grand canonical Hessian $\underline{\mathscr{H}}^{*-1}$ by matrix inversion of $\underline{\mathscr{H}}^*$.

- Calculate the geometric capacitance contribution by:

$$C^*_{\mathrm{geom}} = {C^*_{\mathrm{el}}}^2 \sum_{i,j} \mathscr{H}^{*\,-1}_{i,j} \frac{\partial U}{\partial r_i}^* \frac{\partial U}{\partial r_j}^* \quad .$$

Similarly, we could use potentiostated grand canonical calculations, directly calculate $\underline{\mathscr{H}}^*$ and $\overrightarrow{\nabla} q^*$ (analogous to $\underline{H}^*$ and $\overrightarrow{\nabla} U^*$ above), and use Eq. 9 in the main text. Note however, that in general, one has to use a very high potential-accuracy for the potentiostat, if one wants to determine small contributions to $\underline{\mathscr{H}}^*$ and $\overrightarrow{\nabla} q^*$ accurately enough. A detailed analysis of Hessians and geometric capacitances for the considered states and for the present system are reported below in Sections 7

and 9 of the SI.

One might wonder, why we do not provide a simple closed formula for the geometric capacitance derived from canonical properties only. The reason is that the inverse of the grand canonical Hessian expressed in canonical properties yields a rather complicated expression. We provide a closed formula and an extensive derivation and discussion in Ref. 13.

Electronic + geometric (only reaction path) capacitance approximation (EξGC) For deriving the capacitance in the EξGC approximation along the reaction path at the TS (using purely canonical calculations), first compute the electronic capacitance as described in the EC approximation above. Then perform the following steps using the images along the reaction energy path obtained from the dyNEB at charge q_0:

- Interpolate the canonical energy $E(\xi)$ along the reaction path ξ using a cubic Hermite spline taking into account the projected forces (e.g. as implemented in the ASE[1] NEB module).
- Obtain the position of the transition state ξ^{TS} along the reaction path ξ.
- Interpolate the electrode potential $U(\xi)$ along the reaction path ξ in a smooth way (e.g. a cubic spline).
- Fit a parabola ($a\xi^2 + b\xi + c$) to $E(\xi)$ in the vicinity of the transition state, e.g. in the vicinity of ξ^{TS}. The obtained curvature of the parabola yields $H_{\xi,\xi}^{\mathrm{TS}} = 2a$.
- Fit a linear function ($m\xi + n$) to $U(\xi)$ in the vicinity of the transition state, e.g. in the vicinity of ξ^{TS}. The obtained slope of the linear fit yields $\frac{\partial U}{\partial \xi}^{\mathrm{TS}} = m$.
- Calculate the corresponding value of the grand canonical Hessian via $\mathscr{H}_{\xi,\xi}^{\mathrm{TS}} = H_{\xi,\xi}^{\mathrm{TS}} - C_{\mathrm{el}} \left(\frac{\partial U}{\partial \xi}^{\mathrm{TS}}\right)^2$ and invert it: $\mathscr{H}_{\xi,\xi}^{\mathrm{TS}^{-1}} = \frac{1}{\mathscr{H}_{\xi,\xi}^{\mathrm{TS}}}$.
- Obtain the geometric capacitance contribution via $C^*_{\mathrm{geom},\xi} = \frac{C_{\mathrm{el}}^2 \left(\frac{\partial U}{\partial \xi}^{\mathrm{TS}}\right)^2}{\mathscr{H}_{\xi,\xi}^{\mathrm{TS}}}$.

Again, we could also perform these steps on the basis of a grand canonical NEB calculation, directly yielding $\mathscr{H}^{\mathrm{TS}}_{\xi,\xi}$ and $\frac{\partial q}{\partial \xi}$ (similar as the canonical NEB calculation yields $H^{\mathrm{TS}}_{\xi,\xi}$ and $\frac{\partial U}{\partial \xi}$) that we could insert into Eq. 15 of the main text. At least for the studied system, both approaches yield essentially identical results, as shown in Fig. S4. Note, however, that in general the normal modes (and hence the direction of the reaction path at the transition state) can slightly deviate in the canonical and the grand canonical ensemble since the Hessians differ (cf. Ref. 13 and Section 6 below for the math and Section 7, where we analyze in more detail normal modes and Hessians for the TS.).

4 Absolute one-dimensional grand canonical PES along the reaction path

In the following we shortly want to outline how we created Fig. 1 of the main text which illustrates the one-dimensional grand canonical PES along the reaction path.

- Perform a constant charge NEB calculation at $q = 0$.
- Freeze the obtained geometries, the NEB *images*, along the reaction path and perform several charged single point calculations for every image. Essentially evaluate the NEB without further geometric optimization at various charges $q_i = -1.111, -0.999, ..., 0.999, 1.111$. For all images determine their positions ξ_j along the reaction path ξ. Obtain the canonical energies E_i, the forces $F_i^{\|}$ projected onto the reaction path, and the electrode potentials U_i for the various q_i.
- In order to take into account the information encoded in the projected forces, for every q_i fit the canonical energy along the reaction path using a cubic Hermite spline taking into account the projected forces as implemented in the ASE[1] NEB module.
- For every q_i evaluate the just derived canonical energy fit on a fine grid along the reaction path. Note that incorporating the projected forces at the various excess charges q_i leads to a

slight shift of the transition state. Essentially it is the canonical shift that we derive in Ref. 13 (considering only one spatial dimension). Note, that, hence, we take into account only the geometric shift in one dimension, neglecting a possible shift perpendicular to the $q = 0$ reaction path that might results from other $q_i \neq 0$. The limitation to the purely one-dimensional case is why we consider this more a qualitative illustration.

- Perform a two-dimensional spline interpolation of E in dependence of q and ξ. This essentially yields the canonical PES $E(q, \xi)$.
- Perform a two-dimensional spline interpolation of U in dependence of q and ξ yielding $U(q, \xi)$.
- Define a function $q(U, \xi)$ that inverts $U(q, \xi)$ at every ξ.
- Define a function returning the grand canonical energy as $\mathscr{E}(U, \xi) = E(q(U, \xi), \xi) - q(U, \xi)U$. This is the grand canonical PES as plotted in Fig. 1 of the main text.
- For the constant potential lines, evaluate $\mathscr{E}(U, \xi)$ at constant U. The transition state position is where $\mathscr{E}$ reaches is maximum.
- For the constant charge lines evaluate $\mathscr{E}(U(q, \xi), \xi))$ at constant q. Since we are at constant charge conditions along this path, the transition state position is where where $E(q, \xi)$ reaches its maximum (i.e. not where $\mathscr{E}(U(q, \xi), \xi))$ reaches its maximum). While this might seem counter-intuitive at the first glance, it illustrates that a stationary point of the grand canonical PES at constant potential conditions is a stationary point in the canonical PES at constant charge conditions.

5 Including the reference state

We want to introduce one additional state, that we denote as the 'reference state' RS, which essentially is identical to the initial state, however with the H^+ that takes part in the reaction

considered in the bulk electrolyte and the corresponding electron e^- in the external electron bath.[a]

For simplicity, in the main text of this work we focused on states of same composition (IS, TS, and FS), i.e. we do not consider a change in the number of atoms. As shown in previous work[16–19], we can incorporate the effect of compositional changes by accounting for changes in the number of (possibly charged) adsorbates N_a (i.e. non-substrate atoms) and similarly for changes in the number of (neutral) substrate atoms N_s via the corresponding (electro-)chemical potentials $\tilde{\mu}_a$ and μ_s. We will in the following, however, assume a constant number of substrate atoms and hence ignore their contribution. For a derivation including the energetics of substrate atoms see e.g. Refs. 16 and 17.

Before we continue, note that the absolute excess charge q is not uniquely defined, i.e. in principle we could assign q to the total number of electrons of the simulated system. However, it is more sensible in an electrochemical context to consider q as the net excess charge of the system. Essentially this means, we assign $q = 0$ to the charge-neutral state of the surface, where there is no counter charge present in the (continuum) electrolyte. With this in mind, if we add a charged species, e.g. an isolated ion with charge q_a, to the system, we similarly have to add a compensating electronic counter charge $-q_a$ in order to obtain an overall charge neutral interface again, i.e. the absolute electronic excess charge $q_{\mathrm{abs}} = q - N_a q_a$ has to compensate any charges of explicit ions *and* the charge of the electrolyte. The energy of N_a species of type a together with their respective compensating electrons in the respective bulk reservoirs is hence given by:

$$E_a(U) = N_a \tilde{\mu}_a + (-q_a) N_a U \quad . \tag{2}$$

Note that this way of counting electrons does *not* couple ionic and electronic degrees of freedom since the net excess charge q is still an independent variable.

Now, we want to compare the energy of the RS state to the energies of IS, TS, and FS. To do so, we can add the contribution $E_a(U)$ from Eq. 2 to the grand canonical energy of the reference state $\mathscr{E}^{\mathrm{RS}}(U)$.

[a]The RS is sometimes also called the 'true initial state' in contrast to the 'pseudo initial state' that we just denote as initial state.[14,15]

Instead of computing the electrochemical potential of a proton H^+ in the bulk electrolyte, we make use of the Computational Hydrogen Electrode approach[20] where the energy of (H^+ + e^-) in their respective reservoirs at an arbitrary absolute potential U (Eq. 2) and at pH = 0 is given as:[16]

$$E_{\mathrm{H^+ + e^-}}(U) = \frac{1}{2}\mu(\mathrm{H_2(g)}) + 4.44\mathrm{eV} - eU \quad ,$$

leveraging that the Standard Hydrogen Electrode (SHE) scale is related to the absolute electrode potential via:[21]

$$U_{\mathrm{SHE}} = 4.44\mathrm{V} \quad .$$

We derive $\mu(\mathrm{H_2(g)})$ by performing a vacuum calculation and computing the free energy in gas phase at a temperature of 300 K and a pressure of 1 bar via the ideal gas approximation as implemented in the thermochemistry module of ASE[1]. Note, that in this work we only discuss the results at pH = 0, however, we could also include pH effects by considering the Reversible Hydrogen Electrode potential scale (RHE) similar to the procedure in Ref. 16. Note, that instead of adding $E_{\mathrm{H^+ + e^-}}(U)$ to the grand canonical energy of the reference state, we could similarly subtract it from IS, TS, and FS. Again, the specific choice is arbitrary and – as long as only considering relative energies – irrelevant. For the absolute energetics we show the additive referencing in Fig. S2 and the subtractive referencing in Fig. S3, respectively. Note, that we could equally consider the 'excess energy' of every state, i.e. the energy cost of creating the entire system from suitable reservoirs (see Ref. 17 for more details).

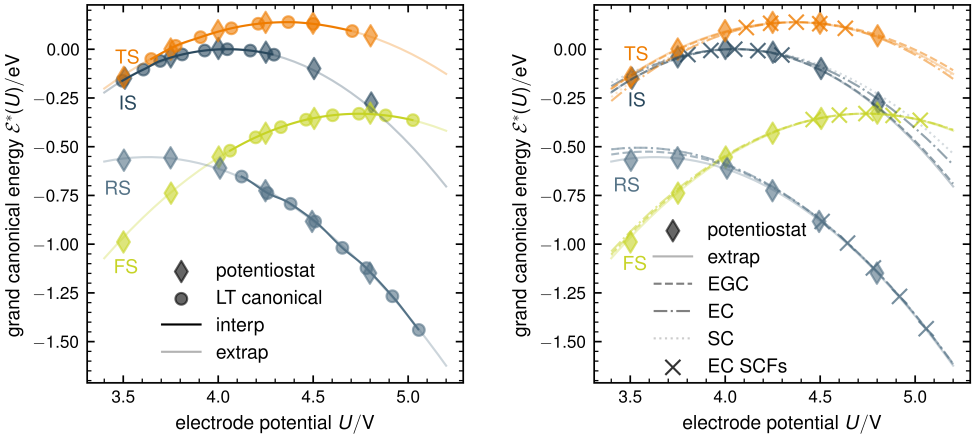

Figure S2: Grand canonical energetics of RS, IS, TS, and FS as a function of absolute electrode potential derived using the various methods discussed in the main text. For the reference state, we add the energy $E_{H^+ + e^-}(U)$ of H^+ + e^- in their respective bulk reservoirs.

Left: Inter-/Extrapolated grand canonical energies derived from eight constant charge climbing image NEBs for $q = -0.555, -0, 444, ..., 0.222e$ (from left to right) in comparison with potentiostat results (diamonds). Cubic Hermite spline interpolation as solid line, quadratic extrapolation as dashed lines.

Right: Single NEB calculation based approaches – SC, EC, and EGC approximation (dotted, dash-dotted, and dashed lines) – in comparison to the extrapolated (light solid lines) and reference potentiostat results (diamonds). The electronic SCF calculations used for determining the electronic capacitance of the EC approach are shown as crosses.

Note, that the reference state RS without the additional adsorbate related term $E_{H^+ + e^-}(U)$ had its PZC roughly around the PZC of the FS.

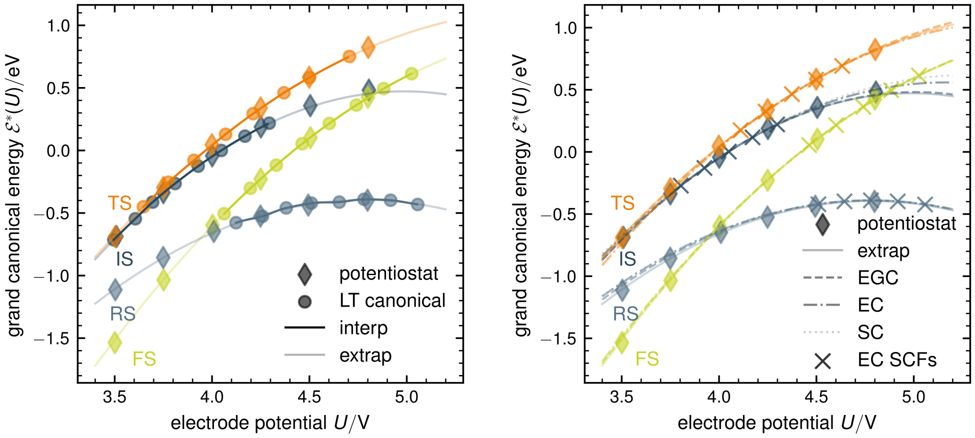

Figure S3: Same as Fig. S2, but subtracting the energy $E_{H^+ + e^-}(U)$ of H^+ + e^- in their respective bulk reservoirs from IS, TS, and FS instead of adding it to the RS.

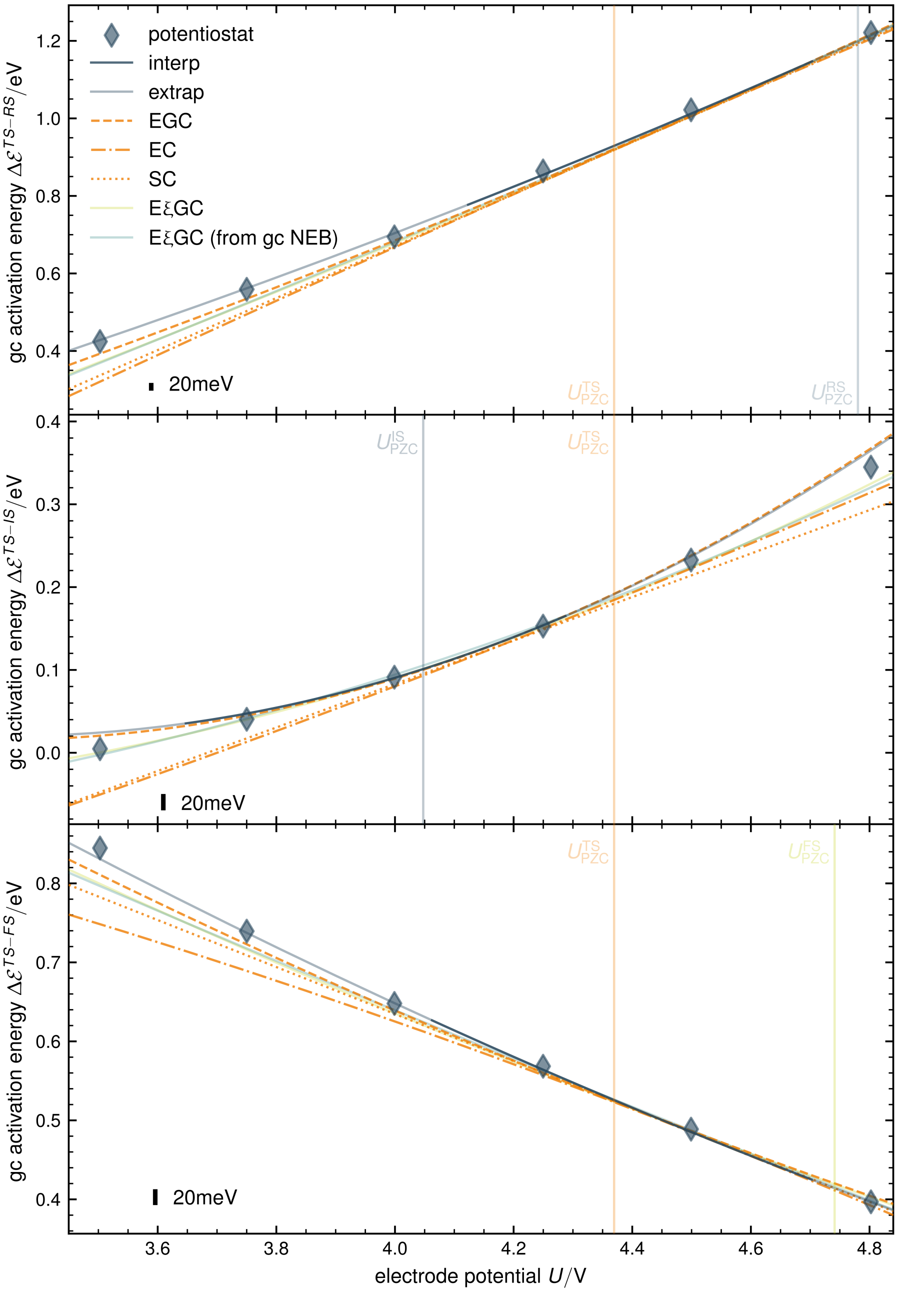

Figure S4: Comparison of kinetic activation barriers as a function of the absolute electrode potential. Forward barrier with respect to the RS (top panel) and with respect to IS (middle panel), and reverse barrier with respect to FS (bottom panel). Multiple-NEB calculation based methods (blue solid lines): interpolation (dark), extrapolation (light), both based on parabolic fits. Single-NEB methods (orange lines): EGC (electronic + geometric, dashed), EC (electronic only, dash-dotted), and SC (single capacitance, dotted). Reference potentiostat data is shown as dark blue diamonds. Note the strong influence of geometric effects, i.e. the difference between EGC (dashed) and EC approximation (dash-dotted) which are to large extent originating from contributions along the reaction path ξ (EξGC, bright green lines). Also shown here is the EξGC approximation derived from a potentiostated grand canonical NEB calculation (gc NEB) as dark green lines which yields the same result. The PZCs of the relevant states U^{*}_{PZC} are drawn as vertical lines.

6 Derivation of the geometric capacitance and the grand canonical Hessian

In this section we summarize the derivation of the central equations Eqs. 9-11 of the main text, which are extensively explained, derived, and discussed in Ref. 13.

We consider the grand canonical energy $\mathscr{E}$ a function of electrode potential U and system geometry $\vec{r}$ of the system: $\mathscr{E}(U,\vec{r})$. We denote the potential dependent 3N-dimensional atomic coordinates of a specific geometrically stationary point, e.g. IS, TS, or FS, as $\vec{r}^*(U)$, which can be interpreted as a smooth path in the potential window where the stationary point is energetically stable. The path is defined by the stationarity criterion $\vec{\mathscr{F}}^* = \vec{\mathscr{F}}(U,\vec{r}^*(U)) = 0 = \text{const.}$[13]

To increase clarity, for partial derivatives we explicitly indicate quantities held constant, e.g. by writing $\left.\frac{\partial X}{\partial r_i}\right|_U$ for the spatial derivative of a quantity X in direction of coordinate i at constant potential U. Note, that $\vec{r}^*(U)$ is only a function of one variable, the electrode potential U, so partial and total derivative are identical.

The total derivative of $\mathscr{E}$ with respect to electrode potential U for a stationary point is given by the excess charge q:[13]

$$\frac{\mathrm{d}}{\mathrm{d}U}\mathscr{E}(U,\vec{r}^*(U)) = \left.\frac{\partial \mathscr{E}}{\partial U}\right|_r (U,\vec{r}^*(U)) + \sum_i \left.\frac{\partial \mathscr{E}}{\partial r_i}\right|_U (U,\vec{r}^*(U)) \cdot \frac{\mathrm{d}r_i^*}{\mathrm{d}U}(U) = -q(U,\vec{r}^*(U)) \quad , \tag{3}$$

using the chain rule, the stationarity condition $\frac{\partial \mathscr{E}}{\partial r_i}(U,\vec{r}^*(U)) = -\mathscr{F}_i(U,\vec{r}^*(U)) = 0$, and using that $\left.\frac{\partial \mathscr{E}}{\partial U}\right|_r (U,\vec{r}) = -q(U,\vec{r})$ defines the excess charge of the system.

Furthermore, since the stationarity is valid along the entire path $\vec{r}^*$, i.e. $\vec{\mathscr{F}}^* = 0 = \text{const.}$ and hence $\frac{\mathrm{d}}{\mathrm{d}U}\vec{\mathscr{F}}(U,\vec{r}^*(U)) = 0$, we can write using the grand canonical Hessian $\underline{H}$ with $\mathscr{H}_{i,j} =$

$\frac{\partial^2 \mathscr{E}}{\partial r_j \partial r_i}\Big|_U (U, \vec{r}) = -\frac{\partial \mathscr{F}_i}{\partial r_j}\Big|_U (U, \vec{r})$ and its inverse $\underline{\underline{\mathscr{H}}}^{-1}(U, \vec{r})$:[13]

$$
\begin{aligned}
0 = \frac{\mathrm{d}}{\mathrm{d}U}\mathscr{F}_i(U, \vec{r}^*(U)) &= \frac{\partial \mathscr{F}_i}{\partial U}\Big|_r (U, \vec{r}^*(U)) + \sum_j \frac{\partial \mathscr{F}_i}{\partial r_j}\Big|_U (U, \vec{r}^*(U)) \cdot \frac{\mathrm{d}r_j^*}{\mathrm{d}U}(U) \\
&= \frac{\partial \mathscr{F}_i}{\partial U}\Big|_r (U, \vec{r}^*(U)) - \sum_j \mathscr{H}_{i,j}(U, \vec{r}^*(U)) \cdot \frac{\mathrm{d}r_j^*}{\mathrm{d}U}(U) \\
\Leftrightarrow \quad \frac{\mathrm{d}r_k^*}{\mathrm{d}U}(U) &= \sum_i \mathscr{H}_{k,i}^{-1}(U, \vec{r}^*(U)) \cdot \frac{\partial \mathscr{F}_i}{\partial U}\Big|_r (U, \vec{r}^*(U)) \quad , \qquad (4)
\end{aligned}
$$

yielding the potential dependent change of the path $\vec{r}^*(U)$.

By differentiating Eq. 3 a second time while inserting Eq. 4) we can derive the total capacitance of the system as the negative total curvature of the grand canonical energy of a stationary point with respect to electrode potential:[13]

$$
\begin{aligned}
C_{\mathrm{total}}^*(U) &= -\frac{\mathrm{d}^2}{\mathrm{d}U^2}\mathscr{E}(U, \vec{r}^*(U)) \\
&= \frac{\mathrm{d}}{\mathrm{d}U} q(U, \vec{r}^*(U)) \\
&= \frac{\partial q}{\partial U}\Big|_r (U, \vec{r}^*(U)) + \sum_i \frac{\partial q}{\partial r_i}\Big|_U (U, \vec{r}^*(U)) \cdot \frac{\mathrm{d}r_i^*}{\mathrm{d}U}(U) \\
&= C_{\mathrm{el}}(U, \vec{r}^*(U)) + \sum_{i,j} \frac{\partial q}{\partial r_i}(U, \vec{r}^*(U)) \cdot \mathscr{H}_{i,j}^{-1}(U, \vec{r}^*(U)) \cdot \frac{\partial \mathscr{F}_j}{\partial U}(U, \vec{r}^*(U)) \\
&= C_{\mathrm{el}}(U, \vec{r}^*(U)) + \sum_{i,j} \frac{\partial q}{\partial r_i}(U, \vec{r}^*(U)) \cdot \mathscr{H}_{i,j}^{-1}(U, \vec{r}^*(U)) \cdot \frac{\partial q}{\partial r_j}(U, \vec{r}^*(U)) \qquad (5)
\end{aligned}
$$

where we used our definition of the purely electronic capacitance $C_{\mathrm{el}}(U, \vec{r}) = \frac{\partial q}{\partial U}\Big|_r (U, \vec{r}) = -\frac{\partial^2}{\partial U^2}\Big|_r \mathscr{E}(U, \vec{r})$ and in the last step Schwarz's theorem:

$$
\frac{\partial \mathscr{F}_i}{\partial U}\Big|_r (U, \vec{r}) = -\frac{\partial}{\partial U}\Big|_r \frac{\partial}{\partial r_i}\Big|_U \mathscr{E}(U, \vec{r}) = -\frac{\partial}{\partial r_i}\Big|_U \frac{\partial}{\partial U}\Big|_r \mathscr{E}(U, \vec{r}) = \frac{\partial q}{\partial r_i}\Big|_U (U, \vec{r}) \quad . \qquad (6)
$$

By defining the geometric capacitance as $C_{\mathrm{geom}}^* = C_{\mathrm{total}}^* - C_{\mathrm{el}}^*$ and by incorporating the dependence on the potential-dependent positions of the stationary state $\vec{r}^*(U)$, i.e. by rewriting a quantity X

that depends on $(U, \overrightarrow{r}^*(U))$ as a new quantity "$X^*(U)$", Eq. 5 reads in matrix notation:[13]

$$C^*_{\text{geom}}(U) = C^*_{\text{total}}(U) - C^*_{\text{el}}(U) = \overrightarrow{\nabla} q^{*T}(U) \underline{\mathscr{H}}^{*-1}(U) \overrightarrow{\nabla} q^*(U) \quad , \tag{7}$$

which is essentially Eq. 9 in the main text.

As written in the main text and discussed in Ref. 13, it is possible to express the grand canonical Hessian $\underline{\mathscr{H}}$ in terms of the canonical Hessian H by starting with:

$$\begin{aligned}\mathscr{H}_{i,j}(U,\overrightarrow{r}) &= \frac{\partial^2}{\partial r_j \partial r_i}\Big|_U \mathscr{E}(U,\overrightarrow{r}) = \frac{\partial}{\partial r_j}\Big|_U \frac{\partial}{\partial r_i}\Big|_U \mathscr{E}(U,\overrightarrow{r}) \\ &= -\frac{\partial}{\partial r_j}\Big|_U \mathscr{F}_i(U,\overrightarrow{r}) \quad .\end{aligned}$$

Using the equivalence of grand canonical and canonical forces $\mathscr{F}_i = F_i$, i.e. $\mathscr{F}_i(U,\overrightarrow{r}) = F_i(q(U,\overrightarrow{r}),\overrightarrow{r})$, which directly follows from the relation between grand canonical energy $\mathscr{E}$ and canonical energy E given by the Legendre transform $\mathscr{E}(U,\overrightarrow{r}) = E(q(U,\overrightarrow{r}),\overrightarrow{r}) - q(U,\overrightarrow{r})\cdot U$,[13,15] this yields:[13]

$$\begin{aligned}\mathscr{H}_{i,j}(U,\overrightarrow{r}) = &-\frac{\partial}{\partial r_j}\Big|_U F_i(q(U,\overrightarrow{r}),\overrightarrow{r}) \\ &= -\frac{\partial}{\partial r_j}\Big|_q F_i(q(U,\overrightarrow{r}),\overrightarrow{r}) - \frac{\partial}{\partial q}\Big|_r F_i(q(U,\overrightarrow{r}),\overrightarrow{r}) \cdot \frac{\partial}{\partial r_j}\Big|_U q(U,\overrightarrow{r}) \\ &= \frac{\partial^2}{\partial r_j \partial r_i}\Big|_q E(q(U,\overrightarrow{r}),\overrightarrow{r}) - \frac{\partial}{\partial q}\Big|_r F_i(q(U,\overrightarrow{r}),\overrightarrow{r}) \cdot \frac{\partial}{\partial r_j}\Big|_U q(U,\overrightarrow{r}) \quad .\end{aligned}$$

Using the definition of the canonical Hessian $H_{i,j}(q,\overrightarrow{r}) = \frac{\partial^2 E}{\partial r_j \partial r_i}\Big|_q (q,\overrightarrow{r})$, the relation of canonical energy, excess charge and electrode potential $\frac{\partial E}{\partial q}\Big|_r (q,\overrightarrow{r}) = U(q,\overrightarrow{r})$ and Schwarz's Theorem:[13]

$$-\frac{\partial F_i}{\partial q}\Big|_r (q,\overrightarrow{r}) = \frac{\partial}{\partial q}\Big|_r \frac{\partial}{\partial r_i}\Big|_q E(q,\overrightarrow{r}) = \frac{\partial}{\partial r_i}\Big|_q \frac{\partial}{\partial q}\Big|_r E(q,\overrightarrow{r}) = \frac{\partial U}{\partial r_i}\Big|_q (q,\overrightarrow{r}) \quad ,$$

this reads:[13]

$$\mathscr{H}_{i,j}(U,\overrightarrow{r})) = H_{i,j}(q(U,\overrightarrow{r}),\overrightarrow{r}) + \tfrac{\partial}{\partial r_i}\Big|_q U(q(U,\overrightarrow{r}),\overrightarrow{r}) \cdot \tfrac{\partial}{\partial r_j}\Big|_U q(U,\overrightarrow{r})$$
$$\Leftrightarrow \quad \underline{\mathscr{H}} = \underline{H} + \overrightarrow{\nabla} U \overrightarrow{\nabla} q^T \quad ,$$

where we used matrix notation and the outer product notation $\overrightarrow{\nabla} U \overrightarrow{\nabla} q^T$ and for readability skipped the dependencies in the last line.

Finally, using that we can express the gradient of the electrode potential $\overrightarrow{\nabla} U$ in terms of $\overrightarrow{\nabla} q$ using the triple product:[13]

$$\frac{\partial}{\partial r_i}\bigg|_U q(U,\overrightarrow{r}) = -\frac{\partial}{\partial U}\bigg|_r q(U,\overrightarrow{r}) \cdot \frac{\partial}{\partial r_i}\bigg|_q U(q(U,\overrightarrow{r}),\overrightarrow{r})$$
$$\Leftrightarrow \quad \overrightarrow{\nabla} q(U,\overrightarrow{r}) = -C_{\mathrm{el}}(U,\overrightarrow{r}) \cdot \overrightarrow{\nabla} U(q(U,\overrightarrow{r}),\overrightarrow{r}) \quad , \tag{8}$$

this reads:[13]

$$\mathscr{H}_{i,j}(U,\overrightarrow{r})) = H_{i,j}(q(U,\overrightarrow{r}),\overrightarrow{r}) - C_{\mathrm{el}}(U,\overrightarrow{r})\, \tfrac{\partial}{\partial r_i}\Big|_q U(q(U,\overrightarrow{r}),\overrightarrow{r}) \cdot \tfrac{\partial}{\partial r_j}\Big|_q U(q(U,\overrightarrow{r}),\overrightarrow{r})$$
$$\Leftrightarrow \quad \underline{\mathscr{H}} = \underline{H} - C_{\mathrm{el}} \overrightarrow{\nabla} U \overrightarrow{\nabla} U^T \quad ,$$

which is Eq. 10 of the main text.

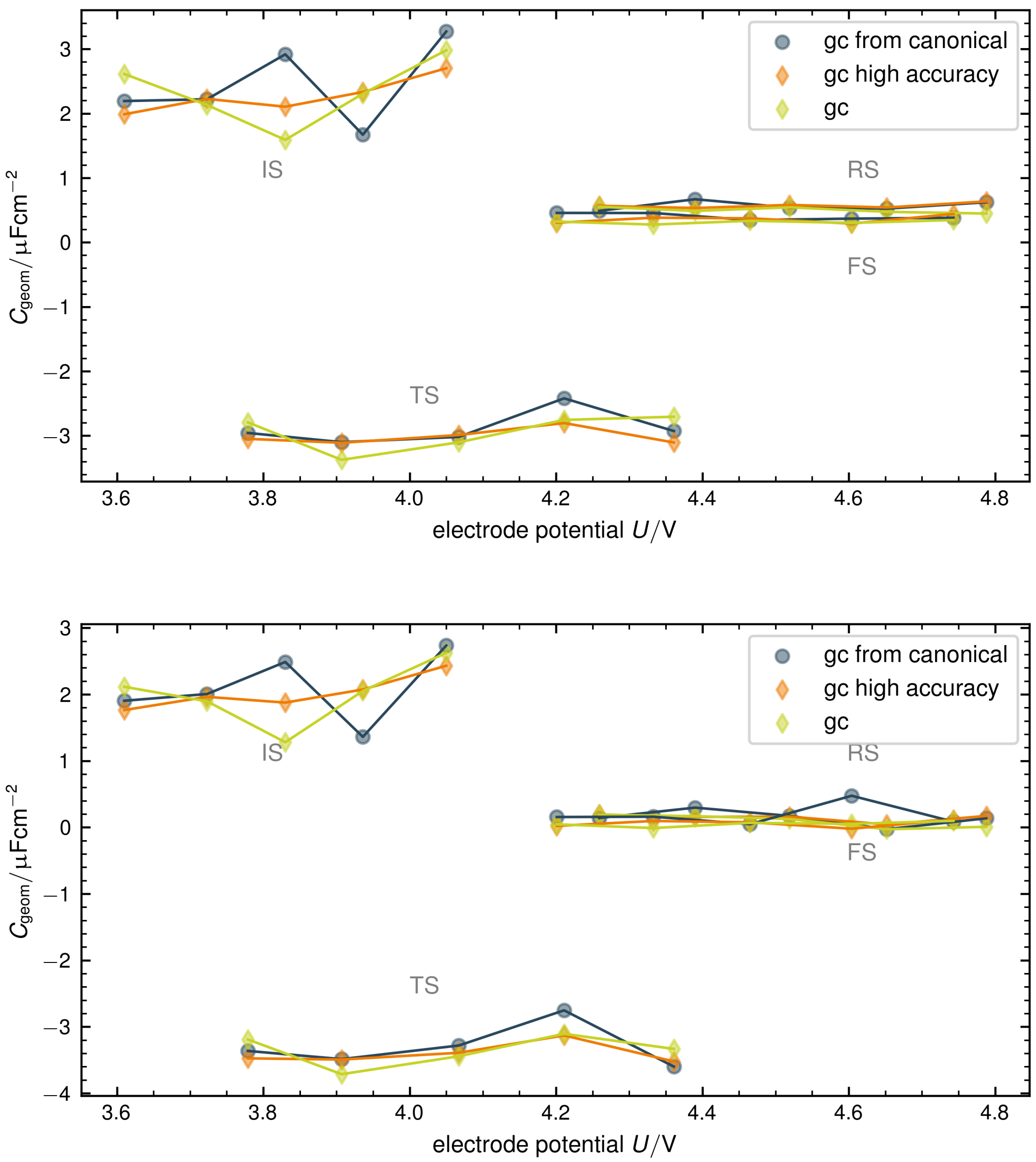

Figure S5: Geometric capacitance contribution C^*_{geom} for IS, TS, FS, and RS in dependence of electrode potential U. Shown are values based on canonical Hessian calculations (dark blue circles) as discussed in the main text and based on direct grand canonical, potentiostated Hessian calculations with normal and very accurate electronic SCF accuracy (light green and orange diamonds) as explained in the text. In the top panel we show the results when including the entire first Au layer and the non-metal atoms in the Hessian calculations and in the bottom panel we show the results when only including the Au atom to which the proton is transferred and the non-metal atoms in the Hessian calculations.

7 Potential dependence of the geometric capacitance

The geometric contribution to the total capacitance $C^*_{\mathrm{geom}} = \overrightarrow{\nabla} q^{*^T} \underline{\mathscr{H}}^{*-1} \overrightarrow{\nabla} q^*$ in principle is a potential dependent quantity (see Ref. 13 and derivation in the previous section). However, we find

it to very good approximation to be constant in the considered potential range as shown in Fig. S5, where we compare the values computed using:

- the procedure explained in the main text, based on canonical quantities only (*gc from canonical*, dark blue circles in Fig. S5),
- the direct computation using a grand-canonical, potentiostated Hessian calculation with a potential tolerance of 1 mV and highly accurate converged electronic SCFs (work function convergence to below 0.1 mV) (*gc high accuracy*, orange diamonds),
- as well as a direct computation using grand-canonical, potentiostated Hessian calculation with default electronic SCF convergence criteria (*gc*, light green diamonds).

The method based on canonical calculations can reproduce the values from the potentiostated grand canonical calculations at comparable accuracy.

As an additional note, it is most important to include the non-metal atoms and only the metal atoms that take part in the reaction in the Hessian calculations (top panel vs bottom panel in Fig. S5).

Note, that the potential dependence might be more significant for more complex systems.

8 Potential dependence of stationary point geometries

The essence of above derivation of the geometric capacitance contribution originates from slight changes in the stationary state geometry as a response to a change in electrode potential $\Delta U = U - U_0$. Certainly the energetic effects are most interesting, however, we can also evaluate the geometric changes themselves. By integrating Eq. 4 from an electrode potential U_0 to $U = U_0 + \Delta U$, assuming a negligible change of $\underline{\mathscr{H}}$ and $\frac{\partial \vec{\mathscr{F}}}{\partial U}\Big|_r$, we obtain the absolute shift $\Delta \vec{r}^*(\Delta U)$ caused by the potential change ΔU accurate to linear order:

$$\Delta r_k^*(\Delta U) = \sum_i \mathscr{H}_{k,i}^{-1}(U_0, \vec{r}^*(U_0)) \cdot \frac{\partial \mathscr{F}_i}{\partial U}\Big|_r (U_0, \vec{r}^*(U_0)) \cdot \Delta U \tag{9}$$

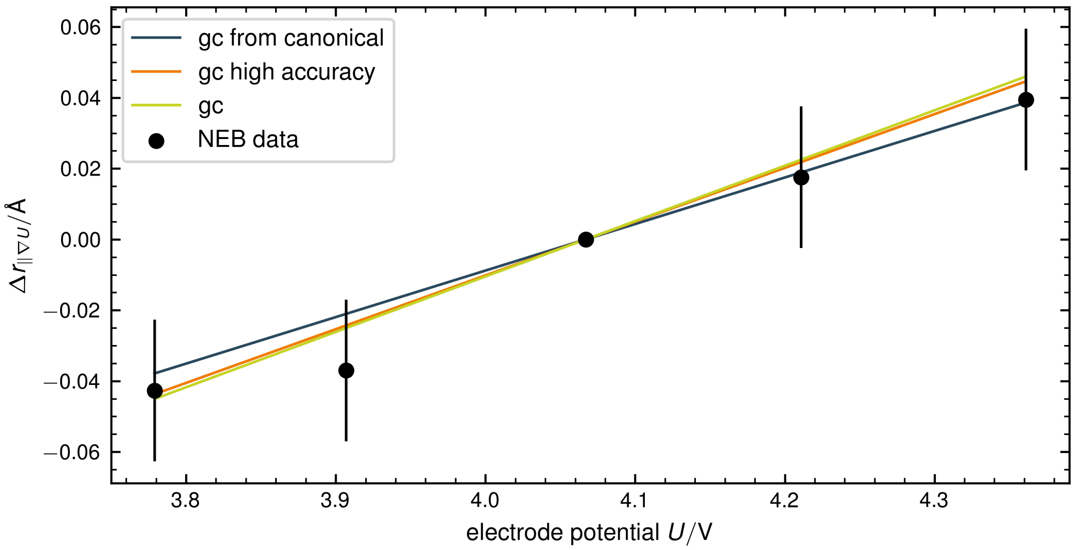

Figure S6: Computed and analytically derived geometric shift $\Delta r^*{}_{\|\nabla U}$ of the transition state along $\overrightarrow{\nabla} U$ for the TS with respect to the geometry at $U_0 = 4.06\,\text{V}$. Shown are the NEB reference points (black) from canonical NEB calculations and the analytical results using Eq. 10 based on high and normal accuracy potentiostatted Hessian calculations (orange and green lines) and derived from purely canonical Hessian calculation data (blue line). The estimated accuracy of the geometries, as discussed in the text, is indicated by black error bars.

that we can also rewrite using Schwarz's Theorem Eq. 6 in terms of $\overrightarrow{\nabla} q$:

$$\Delta r_k^*(\Delta U) = \sum_i \mathscr{H}_{k,i}^{-1}(U_0, \overrightarrow{r}^*(U_0)) \cdot \left.\frac{\partial q}{\partial r_i}\right|_U (U_0, \overrightarrow{r}^*(U_0)) \cdot \Delta U$$

$$\Delta \overrightarrow{r}^*(\Delta U) = \underline{\mathscr{H}}^{-1} \overrightarrow{\nabla} q \Delta U \quad , \tag{10}$$

where we used the matrix notation and dropped the dependence of $\underline{\mathscr{H}}$ and $\overrightarrow{\nabla} q$ on $(U_0, \overrightarrow{r}^*(U_0))$.

Note, that the relevant energetic contributions, i.e. the geometric capacitance contributions $C^*_{\text{geom}}(U)$ (Eq. 7), result solely from the shifts $\Delta r^*{}_{\|\nabla q}$ in direction of $\overrightarrow{\nabla} q$ (or $-\overrightarrow{\nabla} U = \frac{1}{C_{\text{el}}} \overrightarrow{\nabla} q$, see Eq. 8) since $C^*_{\text{geom}} = \overrightarrow{\nabla} q^{*T} \left(\underline{\mathscr{H}}^{*-1} \overrightarrow{\nabla} q^* \right)$.

To validate our results, and to illustrate the geometric shift, we hence compute the shift $\Delta r^*{}_{\|\nabla U}$ for the transition state structure and compare it to the observed shift obtained from actual NEB calculations. Fig. S6 shows the results obtained using the Hessian from the high and normal accuracy potentiostatted calculations (orange and green lines) as well as the ones obtained from

deriving the relevant quantities from purely canonical calculations (blue lines) in comparison to the geometric shifts along $\vec{\nabla} U$ obtained from canonical NEB calculations (black dots) using the geometries of the calculation at $U_0 = 4.06\,\mathrm{V}$ as reference point.

The agreement is remarkable – independent of the underlying Hessian calculations. Note, that the noticeable scatter of the NEB data points is caused by numerical noise originating from the limited accuracy of structure optimizations / NEB calculations. We can roughly estimate the noise δr_d of a structure optimization with a certain force threshold δF (for the TS structure here: $\delta F = 0.03\,\mathrm{eV/Å}$) along a certain (normalized) direction $\vec{d}$, i.e. in this case $\vec{d} = \frac{1}{|\vec{\nabla} U|}\vec{\nabla} U$. For this we compute the curvature $\mathscr{H}_d$ of the PES by evaluating the grand canonical Hessian $\underline{\mathscr{H}}$ along this direction: $\mathscr{H}_d = \vec{d}^T \underline{\mathscr{H}}\,\vec{d}$, which in our case yields $\mathscr{H}_d = -1.3\,\mathrm{eV/Å}^2$ and use common error propagation considering only the dimension along $\vec{d}$: $\delta F_d = \left|\frac{\partial F_d}{\partial r_d}\right| \delta r_d = |\mathscr{H}_d|\,\delta r_d$. The spatial accuracy of the TS geometry δr_d along $\vec{\nabla} U$, hence is given by $\delta r_d = \frac{\delta F_d}{|\mathscr{H}_d|} = \frac{0.03\,\mathrm{eV/Å}}{1.3\,\mathrm{eV/Å}^2} = 0.02\,\mathrm{Å}$. We indicate this estimated accuracy by black error bars around the NEB data points in Fig. S6 confirming the very good agreement between analytically derived and computed geometric shifts.

9 Potential dependence of normal modes, Hessian eigenvalues, and vibrational energy contributions

In the main text, we focus on the grand canonical potential energy surface $\mathscr{E}$ and discuss its relation to the canonical energy E. Considering that we just showed in the previous sections that the grand canonical Hessian $\underline{\mathscr{H}}$ in principle is a potential dependent property and that it might deviate from the canonical Hessian $\underline{H}$, the interested reader might ask, how large the potential dependence is, how large the deviations between grand canonical and canonical Hessian are, and whether they affect vibrational contributions to the energy, i.e. zero point energy ZPE and entropic contributions.

While a more extensive analysis and discussion will certainly be part of future work, we touch on this subject briefly in the following.

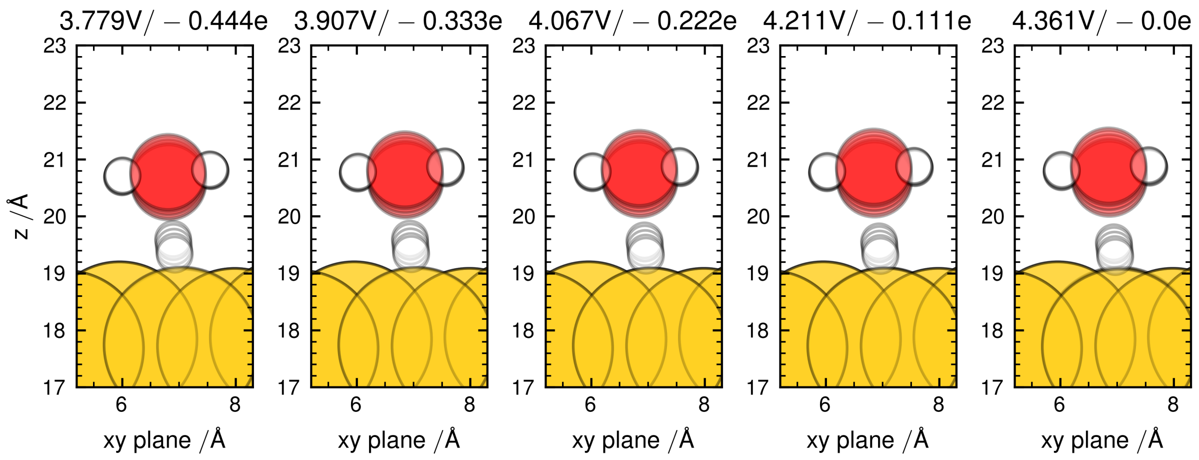

Figure S7: Grand canonical normal modes along the reaction path at the transition state for various electrode potentials U. Movement along the normal mode is shown as an overlay of five frames. Note, that these show the normal mode of the pure Hessians, i.e. the not mass weighted Hessians. While the center positions show a potential dependence, the direction and magnitude of the modes are similar.

Normal modes We illustrate the (grand canonical) normal mode along the reaction path at the transition state at different electrode potentials in Fig. S7. Besides that the center positions, i.e. the geometry of the actual stationary point, slightly changes there are hardly any changes recognizable in the normal mode itself.

Hessian eigenvalues As an example that there might indeed be significant differences between the eigenvalues of the canonical and the grand canonical Hessian, we show the eigenvalues of the normal mode along the reaction path at the transition state and their dependence on electrode potential U in Fig. S8.

Vibrational energy contributions In order to evaluate how the differences in the Hessians as well as their potential dependence affects vibrational free contributions ΔF, we compute according values using the harmonic approximation as implemented in the ASE[1] Thermochemistry module. We consider vibrations of the transferred proton and the neighboring oxygen atom. We show ΔF for both the canonical (dark blue) and the grand-canonical (high accuracy in orange and normal accuracy in green) case in Fig. S9 at a temperature of $T = 300\,\mathrm{K}$. Keep in mind that the eigenmode at the TS along the reaction path (cf Fig. S8 above) does of course not enter the evaluation of ΔF

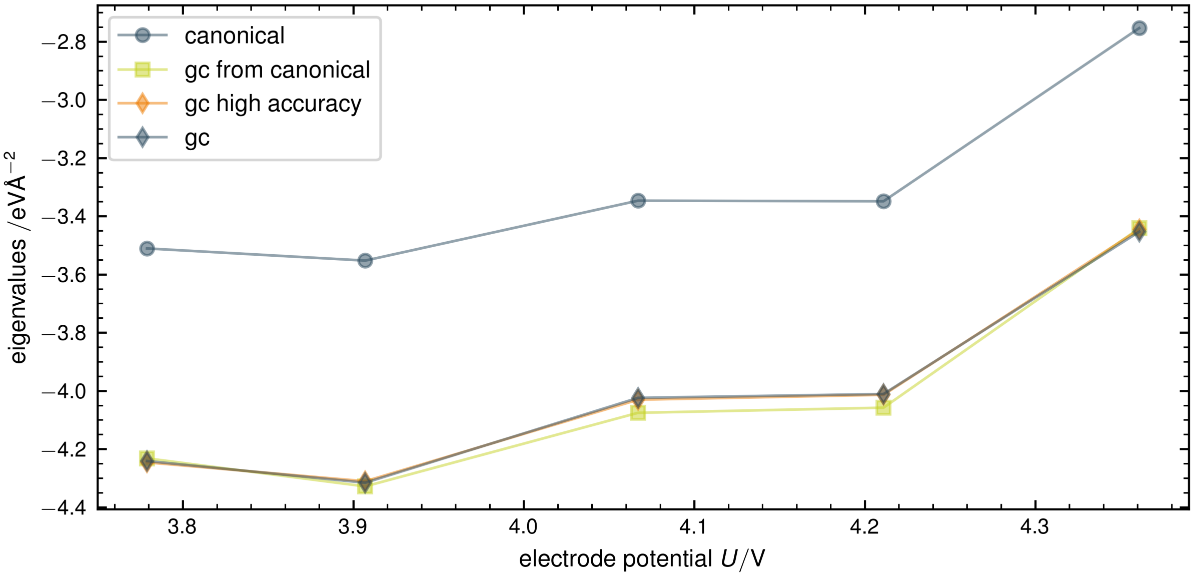

Figure S8: Eigenvalues of the pure, i.e. not mass weighted, canonical and grand canonical Hessians $\underline{H}$ and $\underline{\mathscr{H}}$ for their normal modes along the reaction path at the transition state for various electrode potentials U. Canonical values are shown as blue circles. Grand canonical Hessians $\underline{\mathscr{H}}$ are computed from canonical quantities via Eq. 10 and Eq. 11 of the main text (*gc from canonical*, light green squares), from high accuracy potentiostated grand canonical Hessian calculations as described above (*gc high accuracy*, orange diamonds), and from normal accuracy potentiostated grand canonical Hessian calculations (*gc*, blue diamonds). While the difference between canonical and grand canonical eigenvalues is significant, the three different approaches to compute the grand canonical Hessians yield the same result. All quantities show a similar potential dependence.

but only the vibrational modes orthogonal to it.

While we observe hardly any difference between the canonical and the grand-canonical results (blue vs. green/orange), we can observe a potential-dependence for the IS of around $60\,\mathrm{meV/V}$. However, for the kinetically more relevant states – the FS, the TS and the RS – the potential dependence is almost vanishing. As a result we find that the vibrational free energy contributions at FS, TS and RS can be considered additive constants as mostly done in electrochemical simulations. Note that the vibrational free energy contributions at TS and FS are nearly identical thus suggesting the their inclusion would not alter their relative energetic difference aka the relevant electrochemical barrier. Note also that the difference in ΔF between the FS (water molecule & proton at the interface) and the RS (water molecule without proton at the interface) is approximately $0.12\,\mathrm{eV}$, which is very close to the typical estimates of the vibrational free energy correction of (only) an adsorbed H, e.g. as estimated by considering a zero point energy $\Delta E_{\mathrm{H}}^{\mathrm{ZPE}} = 0.17\,\mathrm{eV}$ in Ref. 22. However, for more

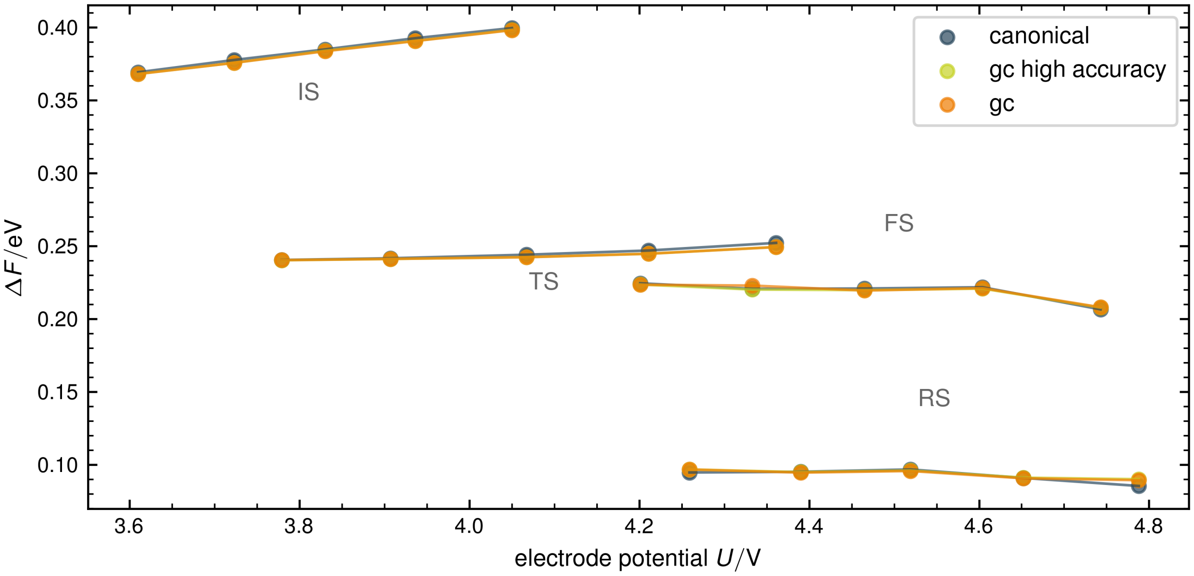

Figure S9: Helmholtz free energy contribution ΔF in dependence of electrode potential U for RS, IS, TS, and FS at $T = 300\,\mathrm{K}$ considering the vibrations of the oxygen and the proton involved in the reaction. The difference between canonical (blue) and grand canonical results (high accuracy orange and normal accuracy green) is negligible. The IS exhibits a slight potential dependence, for the other states the contributions are to good approximation constant. The drop of RS and FS at the highest potentials (close to U_{PZC}, $q = 0\,\mathrm{e}$) originates from a weak rotation-like normal mode of the remaining water molecule, that at other potentials is suppressed by interaction with the charged surface. For the other states this mode is as well suppressed by interaction with the substrate.

detailed analyses and possibly more complex systems one might require a detailed analysis of the potential dependence.

References

(1) Larsen, A. H.; Mortensen, J. J.; Blomqvist, J.; Castelli, I. E.; Christensen, R.; Dułak, M.; Friis, J.; Groves, M. N.; Hammer, B.; Hargus, C.; Hermes, E. D.; Jennings, P. C.; Jensen, P. B.; Kermode, J.; Kitchin, J. R.; Kolsbjerg, E. L.; Kubal, J.; Kaasbjerg, K.; Lysgaard, S.; Maronsson, J. B.; Maxson, T.; Olsen, T.; Pastewka, L.; Peterson, A.; Rostgaard, C.; Schiøtz, J.; Schütt, O.; Strange, M.; Thygesen, K. S.; Vegge, T.; Vilhelmsen, L.; Walter, M.; Zeng, Z.; Jacobsen, K. W. The atomic simulation environment—a Python library for working with atoms. *J. Phys.: Condens. Matter* **2017**, *29*, 273002, DOI: 10.1088/1361-648X/aa680e.

(2) Mortensen, J. J.; Hansen, L. B.; Jacobsen, K. W. Real-space grid implementation of the projector augmented wave method. *Phys. Rev. B* **2005**, *71*, 035109, DOI: 10.1103/PhysRevB.71.035109.

(3) Enkovaara, J.; Rostgaard, C.; Mortensen, J. J.; Chen, J.; Dułak, M.; Ferrighi, L.; Gavnholt, J.; Glinsvad, C.; Haikola, V.; Hansen, H. A.; Kristoffersen, H. H.; Kuisma, M.; Larsen, A. H.; Lehtovaara, L.; Ljungberg, M.; Lopez-Acevedo, O.; Moses, P. G.; Ojanen, J.; Olsen, T.; Petzold, V.; Romero, N. A.; Stausholm-Møller, J.; Strange, M.; Tritsaris, G. A.; Vanin, M.; Walter, M.; Hammer, B.; Häkkinen, H.; Madsen, G. K. H.; Nieminen, R. M.; Nørskov, J. K.; Puska, M.; Rantala, T. T.; Schiøtz, J.; Thygesen, K. S.; Jacobsen, K. W. Electronic structure calculations with GPAW: a real-space implementation of the projector augmented-wave method. *J. Phys. Condens. Matter.* **2010**, *22*, 253202, DOI: 10.1088/0953-8984/22/25/253202.

(4) Kastlunger, G.; Lindgren, P.; Peterson, A. A. Controlled-potential simulation of elementary electrochemical reactions: proton discharge on metal surfaces. *J. Phys. Chem. C* **2018**, *122*, 12771–12781, DOI: 10.1021/acs.jpcc.8b02465.

(5) Wellendorff, J.; Lundgaard, K. T.; Møgelhøj, A.; Petzold, V.; Landis, D. D.; Nørskov, J. K.; Bligaard, T.; Jacobsen, K. W. Density functionals for surface science: Exchange-correlation

model development with Bayesian error estimation. *Phys. Rev. B* **2012**, *85*, 235149, DOI: 10.1103/PhysRevB.85.235149.

(6) Garlyyev, B.; Xue, S.; Watzele, S.; Scieszka, D.; Bandarenka, A. S. Influence of the nature of the alkali metal cations on the electrical double-layer capacitance of model Pt(111) and Au(111) electrodes. *J. Phys. Chem. Lett.* **2018**, *9*, 1927–1930, DOI: 10.1021/acs.jpclett.8b00610.

(7) Kolb, D.; J., S. Surface reconstruction in electrochemistry: Au(100)-(5×20), Au(111)-(1×23) and Au(110)-(1×2). *Electrochimica Acta* **1986**, *31*, 929–936, DOI: 10.1016/0013-4686(86)80005-6.

(8) Trasatti, S.; Lust, E. In *Modern Aspects of Electrochemistry*, 1st ed.; White, R. E., Bockris, J. O., Conway, B. E., Eds.; Springer US, 1999; Vol. 33; pp 1–215, DOI: 10.1007/b113776.

(9) Mills, G.; Jónsson, H.; Schenter, G. K. Reversible work transition state theory: application to dissociative adsorption of hydrogen. *Surf. Sci.* **1995**, *324*, 305–337, DOI: 10.1016/0039-6028(94)00731-4.

(10) Henkelman, G.; Jónsson, H. Improved tangent estimate in the nudged elastic band method for finding minimum energy paths and saddle points. *J. Chem. Phys.* **2000**, *113*, 9978–9985, DOI: 10.1063/1.1323224.

(11) Lindgren, P.; Kastlunger, G.; Peterson, A. A. Scaled and dynamic optimizations of nudged elastic bands. *J. Chem. Theory Comput.* **2019**, *15*, 5787–5793, DOI: 10.1021/acs.jctc.9b00633.

(12) Henkelman, G.; Uberuaga, B. P.; Jónsson, H. A climbing image nudged elastic band method for finding saddle points and minimum energy paths. *J. Chem. Phys.* **2000**, *113*, 9901–9904, DOI: 10.1063/1.1329672.

(13) Beinlich, S. D.; Kastlunger, G.; Reuter, K.; Hörmann, N. G. A theoretical investigation of the grand- and the canonical potential energy surface: the interplay between electronic and

geometric response at electrified interfaces. *arXiv* **2023**, arXiv:2307.09817v1 [physics.chem–ph], DOI: 10.48550/ARXIV.2307.09817.

(14) Lindgren, P.; Kastlunger, G.; Peterson, A. A. A challenge to the G $\sim$ 0 interpretation of hydrogen evolution. *ACS Catal.* **2020**, *10*, 121–128, DOI: 10.1021/acscatal.9b02799.

(15) Lindgren, P.; Kastlunger, G.; Peterson, A. A. Electrochemistry from the atomic scale, in the electronically grand-canonical ensemble. *J. Phys. Chem.* **2022**, *157*, 180902, DOI: 10.1063/5.0123656.

(16) Hörmann, N. G.; Andreussi, O.; Marzari, N. Grand canonical simulations of electrochemical interfaces in implicit solvation models. *J. Chem. Phys.* **2019**, *150*, 041730, DOI: 10.1063/1.5054580.

(17) Hörmann, N. G.; Marzari, N.; Reuter, K. Electrosorption at metal surfaces from first principles. *npj Comput. Mater.* **2020**, *6*, 1–10, DOI: 10.1038/s41524-020-00394-4.

(18) Hörmann, N. G.; Reuter, K. Thermodynamic cyclic voltammograms based on ab initio calculations: Ag(111) in halide-containing solutions. *J. Chem. Theory Comput.* **2021**, *17*, 1782–1794, DOI: 10.1021/acs.jctc.0c01166.

(19) Hörmann, N. G.; Reuter, K. Thermodynamic cyclic voltammograms: peak positions and shapes. *J. Phys.: Condens. Matter* **2021**, *33*, 264004, DOI: 10.1088/1361-648X/abf7a1.

(20) Nørskov, J. K.; Rossmeisl, J.; Logadottir, A.; Lindqvist, L.; Kitchin, J. R.; Bligaard, T.; Jónsson, H. Origin of the overpotential for oxygen reduction at a fuel-cell cathode. *J. Phys. Chem. B* **2004**, *108*, 17886–17892, DOI: 10.1021/jp047349j.

(21) Trasatti, S. The absolute electrode potential: an explanatory note (recommendations 1986). *Pure Appl. Chem.* **1986**, *58*, 955–966, DOI: 10.1351/pac198658070955.

(22) Nørskov, J. K.; Bligaard, T.; Logadottir, A.; Kitchin, J. R.; Chen, J. G.; Pandelov, S.; Stimming, U. Trends in the exchange current for hydrogen evolution. *J. Electrochem. Soc.* **2005**, *152*, J23, DOI: 10.1149/1.1856988.
